# Chemical Variability of the Essential Oil of *Origanum ehrenbergii* Boiss. from Lebanon, Assessed by Independent Component Analysis (ICA) and Common Component and Specific Weight Analysis (CCSWA)

**DOI:** 10.3390/ijms20051026

**Published:** 2019-02-27

**Authors:** Raviella Zgheib, Marc El-Beyrouthy, Sylvain Chaillou, Naim Ouaini, Douglas N. Rutledge, Didier Stien, Amine Kassouf, Marco Leonti, Marcello Iriti

**Affiliations:** 1 Institut Jean-Pierre Bourgin, AgroParisTech, INRA, Université Paris-Saclay, RD 10, Route de Saint-Cyr, 78026 Versailles, France; raviella-zgheib@hotmail.com (R.Z.); sylvain.chaillou1@gmail.com (S.C.); 2 Holy Spirit University of Kaslik, B.P. 446 Jounieh, Lebanon; naimouaini@usek.edu.lb; 3 UMR Ingénierie Procédés Aliments, AgroParisTech, INRA, Université Paris-Saclay, F-91300 Massy, France; rutledge@agroparistech.fr; 4 Laboratoire de Biodiversité et Biotechnologies Microbiennes (LBBM), Observatoire Océanologique, Sorbonne Universités, UPMC Univ Paris 06, CNRS, 66650 Banyuls-sur-mer, France; didier.stien@cnrs.fr; 5 Department of Chemistry and Biochemistry, Faculty of Sciences II, Lebanese University, 90656 Jdeideth El Matn, Lebanon; aminekassouf@hotmail.com; 6 Department of Biomedical Sciences, University of Cagliari, Via Ospedale 72, 09124 Cagliari, Italy; marcoleonti@aim.com; 7 Department of Agricultural and Enviromental Sciences, Milan State University, via G. Celoria 2, 20133 Milan, Italy

**Keywords:** *Origanum ehrenbergii*, essential oils, independent component analysis, common component and specific weight analysis, chemical variability

## Abstract

*Origanum ehrenbergii* Boiss., an endemic plant to Lebanon, is widely acknowledged in Lebanese traditional medicine. The aim of the present study was to evaluate the influence of the drying method, region, and time of harvest on yield and chemical composition of *O. ehrenbergii* essential oils (EOs). Plants were harvested monthly throughout 2013 and 2014, from two different regions, Aabadiye and Qartaba, then dried using two drying methods: lyophilization and shade-drying at 4 °C. EO was extracted by hydrodistillation and analyzed by GC/MS. GC-MS data, combined with independent component analysis (ICA) and common component and specific weight analysis (CCSWA), showed that drying techniques, region of harvest, and soil composition have no effect on the chemical composition of *O. ehrenbergii* EOs. Of the factors analyzed, only harvesting time affected the EO composition of this species. High and stable amounts of carvacrol, associated with reliable antimicrobial activities, were detected in material harvested between March and October. EOs obtained from plants harvested in Aabadiye in January and February showed high amounts of thymoquinone, related to anti-inflammatory and cytotoxic effects. The use of ICA and CCSWA was proven to be efficient, and allowed the development of a discriminant model for the classification of *O. ehrenbergii* chemotype and the determination of the best harvesting time.

## 1. Introduction

In the Mediterranean Basin, Lebanon falls within a recognized center of plant diversity that is considered a global hotspot, ranking third in the world both in plant diversity and endemism [1]. The present study focuses on *Origanum ehrenbergii* Boiss. (Lamiaceae), endemic to Lebanon, where it is known as “za’atar” and “hachichat al jorh” (wound herb). The herb is traditionally used as a cataplasm to stop bleeding and to heal all kinds of wounds [2].

The herb is popular in Lebanon, and prized by the Lebanese mostly as salad and condiment for various food preparations [3]. Synonymously, “za’atar” is a traditional and frequently consumed Lebanese herbal spice mixture used as a bread overlay prepared with olive oil. Besides the herb of *O. syriacum*, which is the main ingredient, the “za’atar” mixture also contains dried and ground leaves and flowers of *O. ehrenbergii*, sesame seeds (*Sesamum indicum* L., Pedaliaceae), seed coat of sumac (*Rhus coriaria* L., Anacardiaceae), and salt (NaCl).

*Origanum ehrenbergii* grows at altitudes between 300 to 2000 m.a.s.l., and its presence has been recorded in different Lebanese regions [4]. Table S1, illustrating the distribution of this species in Lebanon, is provided in the Supplementary Materials section.

EOs can vary in quality and quantity depending on several factors, including climate (precipitation, temperature), seasonal variations (day length, light exposure, harvest period), soil composition (available micro-nutrients, application of fertilizers), vegetative cycle stage, genetic variation (plant ecotype or variety), geographic location (altitude), stress during growth or maturity, and the post-harvest drying and storage [5,6,7,8,9,10].

Basic knowledge in plant phenology and factors influencing the composition of secondary metabolites is essential for the determination of the optimal harvest period [11]. Therefore, this study aims to contribute to a better knowledge of factors influencing the chemical composition of *O. ehrenbergii* Eos, in order to detect possible chemotypes and for the determination of the optimal harvest time depending on the preferences. The main objective of this study was to qualitatively and quantitatively track the variability of the chemical composition of *O. ehrenbergii* EO from March 2013 until December 2014, taking vegetative growth stage, drying method, and geographical location into account. To the best of our knowledge, no studies dealing with the variation of the EO composition of *O. ehrenbergii* have been published to date. *Origanum ehrenbergii*, endemic to Lebanon and growing in localized populations throughout the country, is treated under the protection act (Decision 1/340 (1996) by the Lebanese Ministry of Agriculture), which regulates its harvest and its commercial exploitation. The sustainable production of *O. ehrenbergii* EO constitutes an important economic resource for a part of the rural population of Lebanon.

## 2. Results and Discussion

This section is divided by subheadings. It should provide a concise and precise description of the experimental results, their interpretation, and the experimental conclusions that can be drawn.

### 2.1. Effect of Time of Harvest, Altitude (m.a.s.l.), Drying Methods and Soil Composition on O. ehrenbergii EO Yield

**Figure 1:**
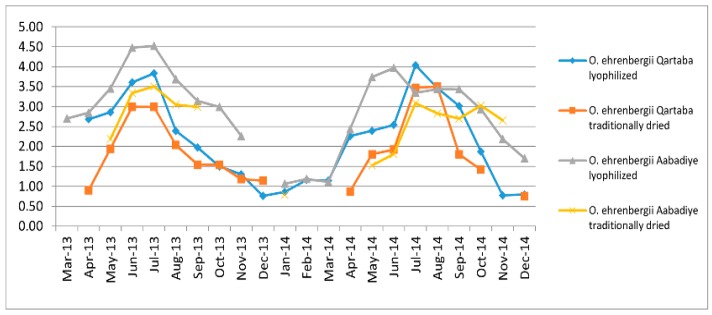
Monthly mean values of essential oil yield (2013–2014) extracted from *Origanum ehrenbergii* harvested in Qartaba and Aabadiye for lyophilized plants and plants shade-dried at 4 °C.

One-way ANOVA indicated significant differences (*p* < 0.05) in EO yield as a function of the growing phase and the date of collection. As can be seen in Figure 1, the mean values of EO yield (mL/g) from *O. ehrenbergii* increased significantly from January to July, and peaked at the flowering stage in June and July. The highest yields were 3.83% in July 2013 and 4.04% in July 2014 for samples harvested from Qartaba and dried by lyophilization, and 4.52% in July 2013 and 3.97% in June 2014 for samples harvested from Aabadiye and dried by lyophilization. Collections made after July showed lower EO contents. These results could be explained by the accumulation of EO during the full-flowering stage, possibly related to ecological pressures such as pathogenic microorganisms (fungi, bacteria virus), herbivory, or the need for attracting pollinators [12,13]. Similar results were found by Rohloff et al. with peppermint (*Mentha x piperita* L.) [14], by Sefidkon et al. with *Satureja rechingeri* Jamzad [15], by Kizil et al. with oregano (*Origanum onites* L.) [16], and by Verdian-rizi with bay leaves (*Laurus nobilis* L.) [17].

In addition, an ANOVA analysis indicated a significant difference in yield as a function of altitude. EO yields obtained from plant material collected at Qartaba (1250 m.a.s.l.) were significantly (*p* < 0.05) lower than those obtained from Aabadiye (600 m.a.s.l.). With increasing altitude, the yields decreased. Table 1 shows the altitudes of the two geographical locations focused on, and the main chemical parameters of the associated soils. Unfortunately, we could not collect samples of *O. ehrenbergii* in all months from the specific sites, due to over-harvesting by local inhabitants. The two sites can be distinguished through a set of parameters (Table 1). In Qartaba (1250 m.a.s.l), the pH and the contents of P_2_O_5_, K_2_O, and nitrogen of the soil were higher, while the organic matter was slightly lower than in Aabadiye (600 m.a.s.l.). It is not clear which factor(s) exerted the strongest influence on EO yield. Although organic matter could, in principle, favor plant growth, the difference in organic matter between the two sites seems too small to have had a significant effect on EO production [18]. Precipitation can also significantly influence EO yield [19]. Since both villages are located on the Mount Lebanon range, exposed to similar quantity of precipitation, this parameter seems not to be responsible [20]. Another possible parameter is the altitude above sea level, which is correlated with significant average temperature differences. In fact, similarly, Avci reported the highest EO yield obtained from *Thymus praecox* ssp. *scorpilii* var. *laniger* samples from the lowest altitude [21], and Baydar reported that *Origanum onites* L. growing at higher altitudes yielded less EO than those from lower altitudes [22]. Additionally, according to Giuliani et al., *Origanum vulgare* L. ssp. *vulgare* EO contents decline with increasing altitude [23]. 

Hence, it is likely that altitude difference is causing yield reduction in this case. Analyses of EO content with plant material collected at different altitudes could provide insights into the causative potential on EO content of this parameter. Further investigations are necessary in order to better understand the effect of altitude and year of harvest on *O. ehrenbergii* EO yield. Lastly, Student’s *t*-tests showed that the two drying methods applied in this study (lyophilization and shade-drying at 4 °C) did not significantly affect EO yields (*p* > 0.05).

### 2.2. Chemical Composition of the Essential Oils

The 94 samples of *O. ehrenbergii* EO obtained by hydrodistillation of dried aerial plant material and analyzed by GC-MS revealed the presence of 54 components, representing 80.4–99.8% of the total EO of the samples of Qartaba and 87.9–99.7% of the total EO of the samples of Aabadiye. Supplementary Tables S2 and S3, provided as Supplementary Materials, show the detected chemical composition of the EOs derived from the 94 *O. ehrenbergii* samples harvested twice a month throughout 2013–2014 from Qartaba and Aabadiye. Compounds were classified into different groups: monoterpene hydrocarbons, oxygenated monoterpenes, sesquiterpene hydrocarbons, oxygenated sesquiterpenes, and other components. Monoterpenes, both monoterpene hydrocarbons and oxygenated monoterpenes were the most frequently represented classes. The main components were: carvacrol (55.3–89.7% in Qartaba; 48.1–88.6% in Aabadiye), *p*-cymene (1.3–16.6% in Qartaba; 1.6–20.9% in Aabadiye), *γ*-terpinene (0.1–4.8% in Qartaba; 0.3–7.1% in Aabadiye), thymoquinone (0.0–2.9% in Qartaba; 0–12.8% in Aabadiye), thymol methyl oxide (1.7–9.3% in Qartaba; 1.1–7.1% in Aabadiye), *β*-bisabolene (0.5–5% in Qartaba; 0.4–4.3% in Aabadiye) and caryophyllene oxide (0.1–2.7% in Qartaba; 0.1–2.9% in Aabadiye).

### 2.3. Evaluation of EO Chemical Variability by Independent Component Analysis (ICA) and Common Component and Specific Weight Analysis (CCSWA)

#### 2.3.1. Effect of Time of Harvest on EO Chemical Composition

● Independent Component Analysis

Carvacrol was biosynthesized consistently in high amounts from March to October (84.2% in Qartaba and 80.2% in Aabadiye for lyophilized samples, and 80.5% in Qartaba and 80.7% in Aabadiye for air-dried samples) (Tables S2 and S3). Its relative proportion was lower in the cold season from November to February, when metabolism slows down.

These conclusions are confirmed by the results of an independent component analysis (ICA) based on harvest time.

**Figure 2:**
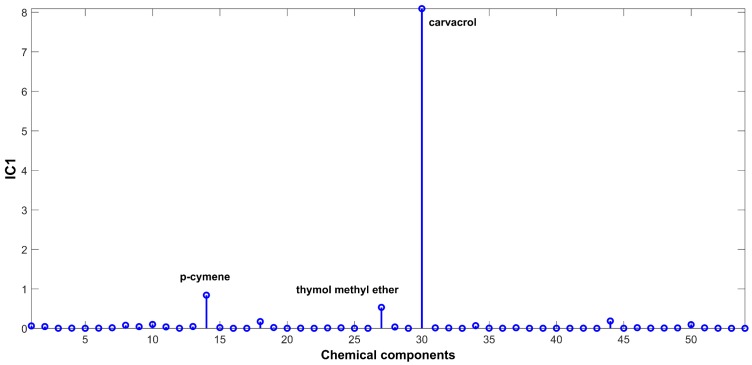
Source signal of IC1 for ICA-DA based on time of harvest.

**Figure 3:**
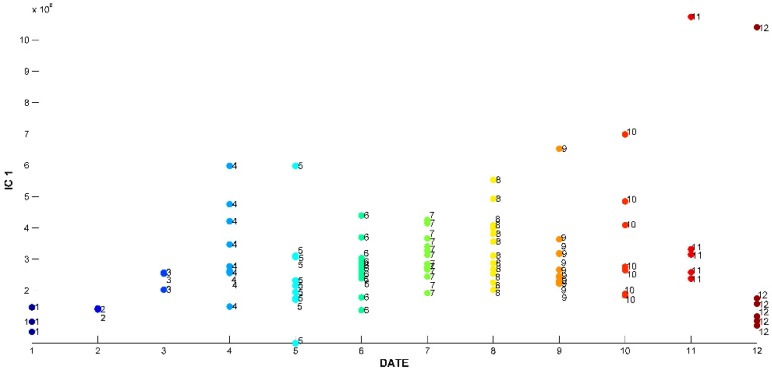
Proportions on IC1 for ICA-DA based on time of harvest. Months numbered from January (1) to December (12).

**Figure 4:**
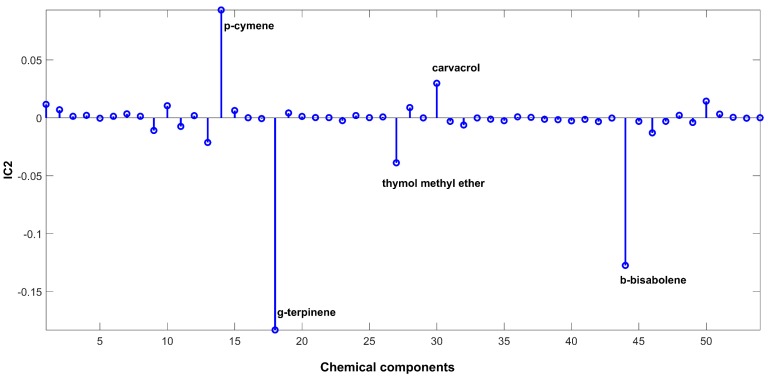
Source signal of IC2 for ICA-DA based on time of harvest.

**Figure 5:**
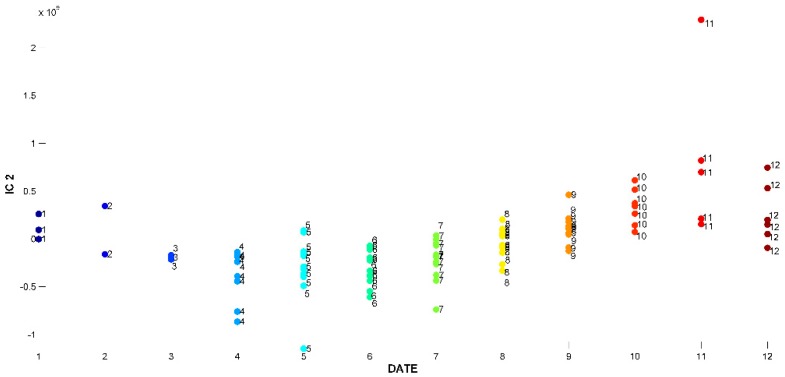
Proportions on IC2 for ICA-DA based on time of harvest. Months numbered from January (1) to December (12).

The signal extracted in the first IC (Figure 2), directly related to signals of chemical compounds extracted from the volatile mixture, is mainly due to carvacrol. The proportions of this IC (Figure 3) are lower in the samples harvested between November and March (months: 1, 2, 3, 11, and 12), and higher in the other months. On the other hand, IC2 has a positive contribution from *p*-cymene, and negative contributions from *γ*-terpinene and *β*-bisabolene (Figure 4). As can be seen in the corresponding proportions plot (Figure 5), the samples harvested between October and February (months: 1, 2, 10, 11, and 12) should therefore contain more *p*-cymene and less *γ*-terpinene and *β*-bisabolene than the samples harvested between March and September (months: 3–9). 

It seems, thus, that the accumulation of *p*-cymene is correlated with low temperatures. Additionally, *O. ehrenbergii* cytochrome P450 monooxygenase, which transforms *p*-cymene into carvacrol, is down-regulated or less active at low temperatures, resulting in an accumulation of *p*-cymene. At higher temperatures, from March to October, the proportion of carvacrol increased and remained stable regardless of the vegetative stage (pre-flowering, flowering, and post-flowering stages). Overall, flowering itself did not significantly change the EO composition of *O. ehrenbergii*. This result stands in contrast to studies conducted on *O. syriacum*, which noted that the content of the main components, thymol and carvacrol, increased considerably during the period of flowering [24]. The particularity of *O. ehrenbergii* also includes, besides being endemic to Lebanon, its carvacrol content, which contributes more than 80% of the total percentage of the EO. This phenolic compound is known for its pronounced broad-spectrum antibacterial and antifungal activity [25,26,27]. 

● Multi-Block Data Analysis

In order to extract additional information from the GC-MS data concerning the effect of the harvest date on the variation of the EO chemical compounds, CCSWA was applied using the ComDim implementation in the SAISIR toolbox [28]. Six common components (CC) were extracted. CC2 and CC4 contained relevant information and demonstrated new separations, which were not clear in the ICA.

**Figure 6:**
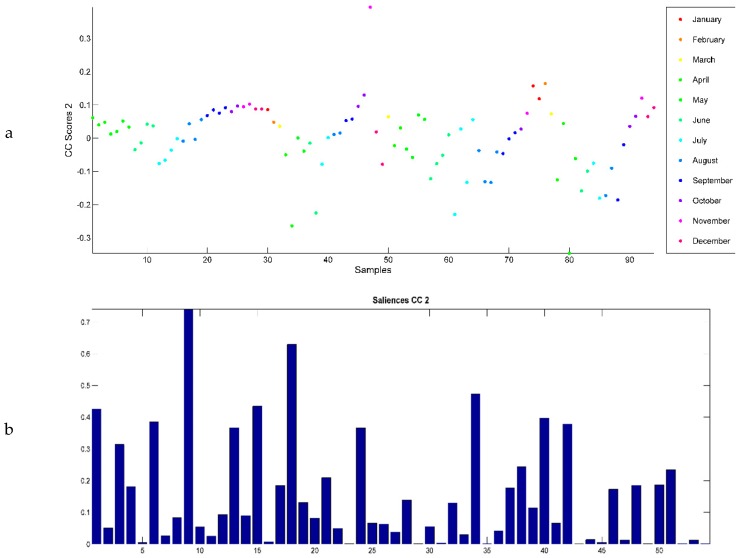

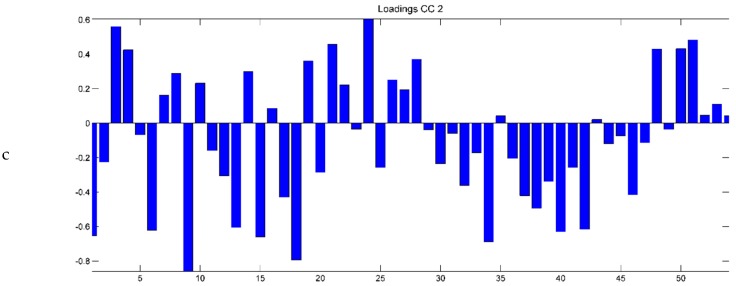
(**a**) CCSWA scores on CC2. (**b**) Saliences corresponding to the 54 chemical compounds of the *O. ehrenbergii* EO according to CC2. (**c**) CCSWA loadings corresponding to the 54 chemical compounds of the *O. ehrenbergii* EO according to CC2.

CC2 (Figure 6a) distinguished samples harvested in June, July, and August from the rest of the samples. The values of the saliences corresponding to CC2 (Figure 6b) showed that the major compounds contributing to CC2 and influencing this discrimination are myrcene (0.74), *γ*-terpinene (0.63), and *β*-caryophyllene (0.473). Looking at the loadings of CC2 (Figure 6c), it can be deduced that the samples harvested in June, July, and August contained more myrcene, *γ*-terpinene, and *β*-caryophyllene than those harvested in all the other months. 

**Figure 7:**
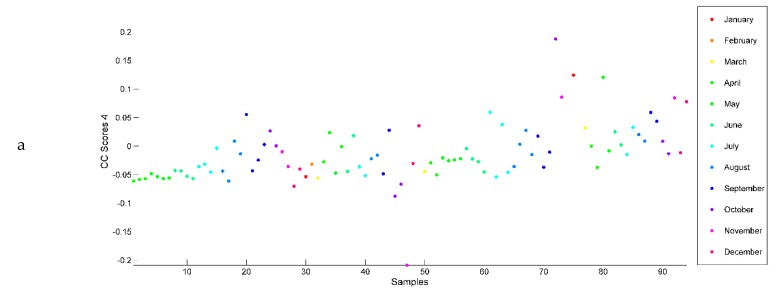

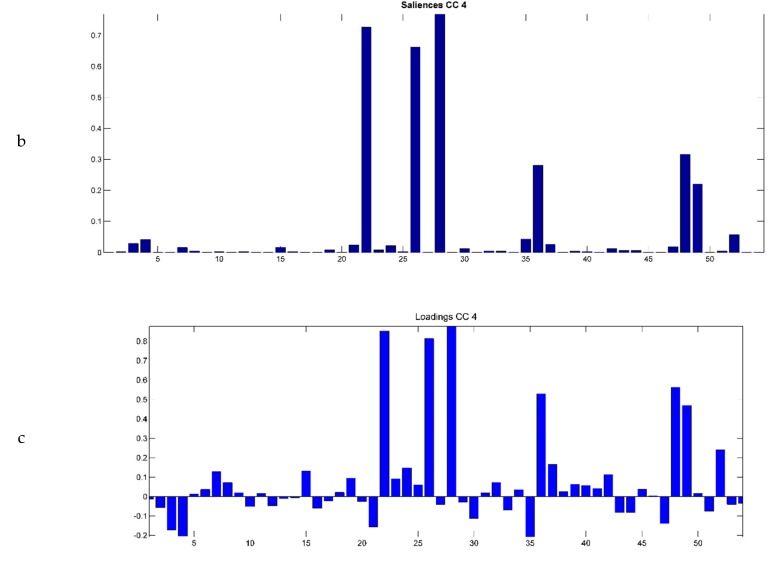
(**a**) Common component and specific weight analysis (CCSWA) scores on CC4. (**b**) Saliences corresponding to the 54 chemical compounds of the *O. ehrenbergii* EO according to CC4. (**c**) CCSWA loadings corresponding to the 54 chemical compounds of the *O. ehrenbergii* EO according to CC4.

CC4 (Figure 7a) showed a slight discrimination of samples harvested in September, October, November, December, January and February from the rest of the samples. According to the saliences of CC4 (Figure 7b), the data tables contributing to this discrimination corresponded mainly to thymoquinone (0.768), linalool (0.726) and *trans*-dihydrocarvone (0.661). The loadings of CC4 (Figure 7c) highlighted that the samples harvested during these months contained more of these three terpenes than those harvested during the rest of the year.

Low levels of thymoquinone, a molecule with promising antioxidant [29] and cytotoxic activities [30,31,32], were observed in Qartaba EO samples (Table S2) (the highest proportions were detected from September to January). The highest amounts of this molecule were recorded in Aabadiye EO samples collected during January 2014, for plant material shade-dried at 4 °C (12.8%), and, in February 2014, for lyophilized material (11.6%) (Table S3). The presence of thymoquinone is thus inversely related to carvacrol. This led us to conclude that, in winter, carvacrol is presumably biotransformed into thymoquinone [33,34].

CCSWA applied to the data set highlighted discriminations that were not extracted in ICA, and emphasized complementary information related to other compounds, given the fact that this method gives an optimal weight to each variable in order to optimize the common dispersion of samples.

In light of this observation, the differences in the accumulation of major biologically active compounds in *O. ehrenbergii* EO associated with the seasonal development of the plant confirm the influence of the physiological stages on EO composition. The observed chemical variability could be due to the interactions between genetic (biotic) factors, climatic and environmental (abiotic) factors, seasonality, age, maturity, and ontogenic stages of the plant [35]. The accumulation of particular terpenes during specific plant developmental stages could also be explained by their ecological roles of defense, securing the plant from predators or abiotic stress [36]. Thus, monitoring plant physiological stages is fundamental for the choice of the best time of harvest.

#### 2.3.2. Effect of Drying Method on EO Chemical Composition

ICA based on drying method showed no difference in the chemical composition of the EO between lyophilization and shade-drying at 4 °C, as similar concentrations of the major constituents were detected.

Monitoring the chemical variation of the EO by CCSWA in relation to specific drying techniques showed that the shade-drying at 4 °C and lyophilization were equivalent, which is in accordance with the result obtained by ICA.

Similarly, in a study focusing on the composition of *T. spicata* EO, it was shown that the drying techniques used did not influence the relative proportion of the three major compounds [37].

#### 2.3.3. Effect of Geographical Location and Soil Composition on EO Chemical Composition

**Figure 8:**
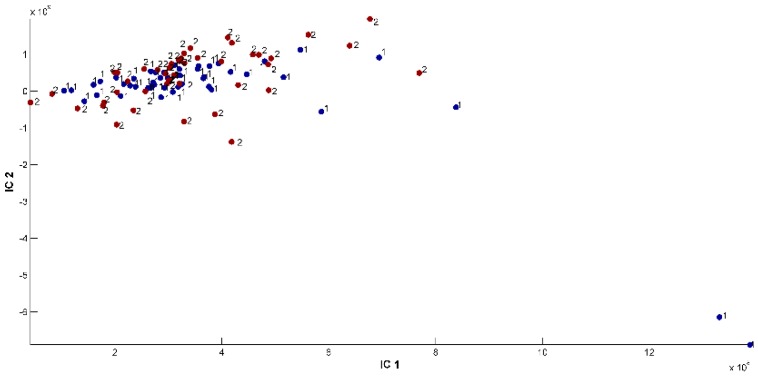
Representation of proportions on the IC1/IC2 plane for ICA-DA based on locality. (**1**) Samples 1 to 49: harvested from Qartaba. (**2**) Samples 50 to 94: harvested from Aabadiye.

**Figure 9:**
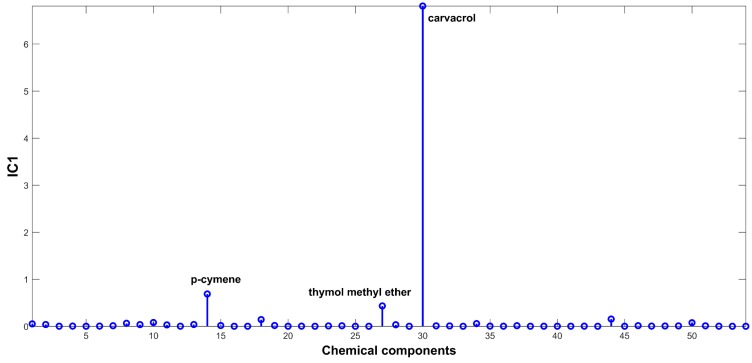
Source signal on IC1.

**Figure 10:**
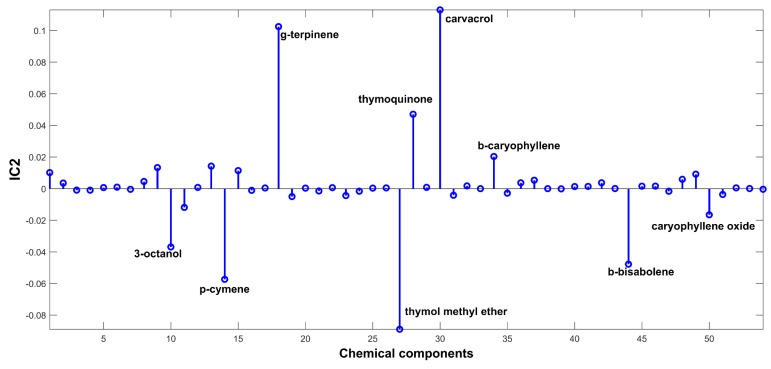
Source signal on IC2.

ICA revealed that the region of harvest did not have a significant impact on *O. ehrenbergii* EO chemical composition. Carvacrol was the main compound identified in the samples collected at Qartaba and Aabadiye, determining the carvacrol chemotype in these two locations. Figure 8 represents the proportions on the IC1/IC2 plane, Figure 9 shows the source signal of IC1, and Figure 10 shows the source signal of IC2.

*O. ehrenbergii* harvested at Qartaba (1250 m.a.s.l.) naturally grows on sandy clay soils while in Aabadiye (600 m.a.s.l.) it grows on sandy silt soils. Our results show that the composition of the oils is not correlated to the place of collection, i.e., the environmental factors associated (such as humidity, altitude, temperature, and climate) and the soil characteristics. It can be concluded that the composition is most probably linked to the botanical variety and genetic factors. A Pearson’s test confirmed this assumption as the correlations of carvacrol, thymoquinone, *p*-cymene, *γ*-terpinene, thymol methyl oxide and *β*-bisabolene with soil characteristics were non-significant (*p* > 0.05).

This finding corroborates the results of Homer et al. [19] and Paula et al. [38], who demonstrated in other species that the chemical composition is controlled by genetic factors.

Furthermore, CCSWA did not highlight any distinction between the oil samples, which is in accordance with the ICA results.

### 2.4. Comparison of EOs of O. ehrenbergii Samples Collected at Qartaba and Aabadiye with Results from other Lebanese Regions

**Figure 11:**
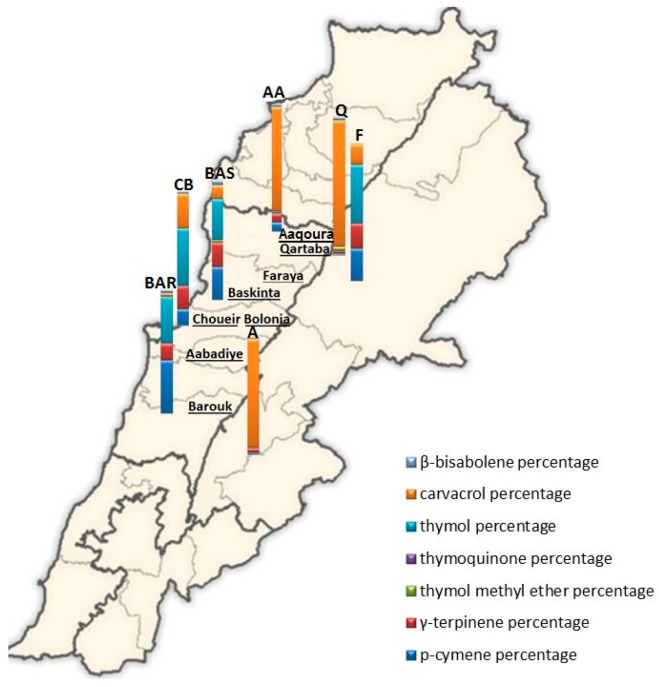
Percentages of *p*-cymene, *γ*-terpinene, thymoquinone, thymol methyl oxide, thymol, carvacrol, and *β*-bisabolene in the total EO of *O. ehrenbergii*. The letters correspond to Lebanese localities where samples were collected (data previously published). BAS: Baskinta [39]; AA: Aaqoura [3]; CB: Choueir Bolonia [3]; BAR: Barouk [40]; F: Faraya [2]; Q: Qartaba (our sample, plant harvested in June 2013 and dried by lyophilization); A: Aabadiye (our sample, plant harvested in June 2013 and dried by lyophilization).

The chemical composition of *O. ehrenbergii* EOs was previously reported for five other collection sites, i.e., from Aaqoura [3], Faraya [2], Baskinta [39], Barouk [40], and Choueir Bolonia [3] (see Figure 11 for comparison with our results). This comparison was conducted based on the values obtained during the full bloom stage, given that the harvest in most of the studies was carried out during this stage.

**Figure 12:**
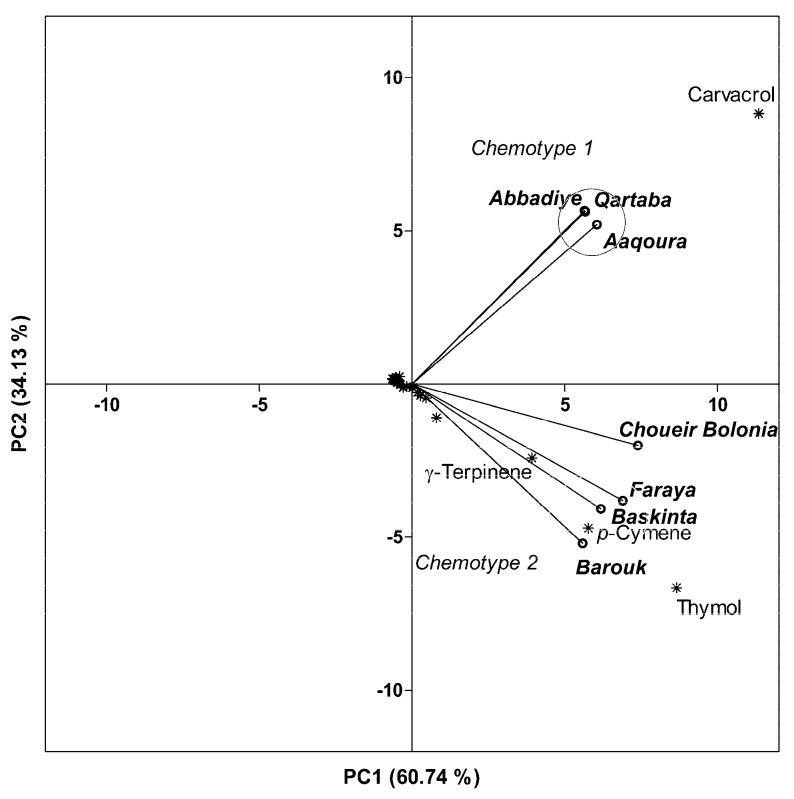
Principal component projection plot of PC1 and PC2 scores and loadings indicating chemotypes within *O. ehrenbergii*, based on GC-MS data of the essential oils.

A principal component analysis (PCA) performed on a combined data matrix gathering percentages of 64 compounds for *O. ehrenbergii* EOs from Aaqoura, Faraya, Baskinta, Barouk, and Choueir Bolonia, as well as our own results from Qartaba and Aabadiye, extracted 94.88% of the total variance in PC1 and PC2. Figure 12 presents a biplot of PC1-PC2 scores and loadings, where a clear separation between the samples from Qartaba, Aabadiye, and Aaqoura and those from Faraya, Baskinta, Barouk, and Choueir Bolonia is visible along PC2.

As can be seen by the proximity of the scores and loadings, the EOs extracted from plants harvested in Qartaba, Aabadiye, and Aaquoura form a group characterized by a high presence of carvacrol. 

The second group consists of *O. ehrenbergii* EOs from Faraya, Baskinta, and Barouk, distinguished by higher amounts of thymol, *p*-cymene, and *γ*-terpinene. 

*O. ehrenbergii* EO from Choueir Bolonia is characterized by an intermediate chemotype: thymol/carvacrol/*p*-cymene/*γ*-terpinene. Whether or not this EO reflects a distinct chemotype is a question that would require the analysis of more samples.

Based on this combined data set, *O. ehrenbergii* EO is characterized by different chemotypes, carvacrol (Chemotype 1) and thymol/*p*-cymene (Chemotype 2), marking the existence of a chemical polymorphism.

## 3. Materials and Methods

### 3.1. Plant Material and Essential Oil Extraction

Aerial parts of *O. ehrenbergii* were collected from a rocky ground near a degraded juniper forest, at Qartaba (34°05’58” N 35°48’46” E/Jbeil District/Mount Lebanon Governorate), at an altitude of 1250 m, and a Pine forest at Aabadiye (33°50’11” N 35°37’13” E/Baabda District/Mount Lebanon Governorate) at an altitude of 600 m. The harvest was conducted twice a month, throughout 2013 and 2014, at all the vegetative stages of plant development. Sampling started from March 1, 2013 and continued until December 1, 2014. Botanical identification of the plant samples was carried out by Dr. Marc El Beyrouthy according to the New Flora of Lebanon and Syria, as described by Mouterde [4]. A voucher specimen of each plant was deposited in the Herbarium of the Faculty of Agricultural and Food Sciences of USEK, Lebanon under the registry numbers MNIII187a for the Qartaba plant and MNIII187b for the Aabadiye plant. The fresh samples (150 g) were either dried in the absence of light in a cold room at 4 °C for one month (shade-drying at 4 °C), or were lyophilized using a Virtis bench top “K” lyophilizer machine. The EOs were obtained by hydrodistillation performed for 3 h using a Clevenger-type apparatus, according to the European Pharmacopoeia [41]. The distilled oils were stored in vials at 4 °C. The composition of the 94 oils (49 obtained from samples harvested at Qartaba and 45 from samples collected at Aabadiye) was determined with GC-MS. Since this plant is over-harvested by local inhabitants, quantities were sometimes insufficient to collect at a constant frequency (twice per month). EO yield, expressed in mL/g, was calculated by measuring the volume of oil extracted per weight of dried/lyophilized plant material. 

### 3.2. Soil Analysis

Three soil samples were collected randomly from each site, at a depth of 0–30 cm. Samples were analyzed at the Lebanese Agricultural Research Institute (LARI) after being dried at room temperature, sieved at 2 mm, and quartered.

### 3.3. Essential Oil Analysis

#### 3.3.1. GC Analyses

Analytical gas chromatography was carried out on a Thermo Electron Corporation gas chromatograph fitted with a flame ionization detector (FID), a non-polar HP-5MS (5% Phenyl Methyl Siloxane capillary column (30 m × 0.25 mm i.d., film thickness 0.25 μm) (Supelco, Sigma-Aldrich, Darmstadt, Germany), and a polar fused-silica HP Innowax polyethylene glycol capillary column (50 m × 0.20 mm i.d., film thickness 0.20 μm) (Supelco, Sigma-Aldrich, Darmstadt, Germany). Helium was the carrier gas (0.8 mL/min). The column temperature was initially set to 35 °C before being gradually increased to 85 °C at 5 °C/min, held for 20 min at 85 °C, raised to 300 °C at 10 °C/min, and finally held for 5 min at 300 °C. Diluted 1 µL samples (1/100 *v*/*v* in pentane) were manually injected at 250 °C in the splitless mode. Flame ionisation detection (FID) was performed at 310 °C.

#### 3.3.2. GC-MS Analyses

The GC-MS analyses were performed using an Agilent gas chromatograph 6890 coupled with a 5975 Mass Detector. The 7683 B auto sampler injected 1 µL of each oil sample diluted in pentane (1/100 *v*/*v*). A fused silica capillary column HP-5MS (5% Phenyl Methyl Siloxane) (30 m × 0.25 mm internal diameter, film thickeness 0.25 µm) (Supelco, Sigma-Aldrich, Darmstadt, Germany) or a fused silica HP Innowax polyethylene glycol capillary column (50 m × 0.20 mm, film thickness 0.20 µm) (Supelco, Sigma-Aldrich, Darmstadt, Germany) was used. Helium was the carrier gas (0.8 mL/min). The oven temperature program was identical to that described above (*cf*. GC Analyses). Mass spectra were recorded at 70 eV with an ion source temperature set at 310 °C and a transfer line heated at 320 °C. Acquisition was recorded in full scan mode (50–400 amu). 

#### 3.3.3. Identifications and Quantifications

Most constituents were identified by comparing mass spectra on both columns with those stored in NIST and Wiley 275 libraries and our home-made library constructed with pure compounds and EOs of known composition (such as the EO of *Rosmarinus officinalis* L. from *Phytosun Aroms*, Plélo, France). The mass spectra were also compared to those from literature [42,43]. A further identification was achieved by comparing their retention indices (RI) on both polar and apolar columns with those from literature [43,44], or with those of standard compounds available in our laboratories, obtained from Sigma-Aldrich (Darmstadt, Germany). Retention indices were determined in relation to a homologous series of *n*-alkanes (C_7_ to C_25_) analyzed under the same GC-MS operating conditions. Relative proportions of oil constituents were calculated from the GC peak areas corrected using the probabilistic quotient normalization (PQN), using the median of all the chromatograms as reference signal for the correction [45].

### 3.4. Essential Oil Analysis

#### 3.4.1. Analysis of Variance

One-way ANOVA test, Pearson’s test, and Student’s *t*-test (SPSS 16.0 software, SPSS Inc., Chicago, IL, USA) were performed to assess whether there were significant variations in the EO yield according to different factors, and in the main EO components according to soil composition. Results were expressed as means ± standard deviation, and considered significantly different at the 0.05 level.

#### 3.4.2. Principal Component Analysis (PCA)

PCA is a multivariate statistical method that calculates a set of new orthogonal axes or variables known as principal components (PCs), which contain most of the variability present in the original variables and which are linear combinations of the original variables. They correspond to directions of greatest dispersion of samples that are projected onto this new dimension. The PCs are mutually uncorrelated, since each successive PC is calculated so as to be orthogonal to all the others [46]. Data treatment was done using XLSTAT 2014.5.03 to examine the discrimination between samples and its chemical constituents.

In fact, one-way analysis of variance (ANOVA), Student’s *t*-test, and Pearson’s test gave valuable information about the correlations between variables. However, no specific information can be extracted, since these techniques were used to determine whether there are any significant differences between individual variables. Furthermore, the vectors calculated by PCA, which may usually denote combinations of diverse phenomena, cannot reveal a physical reality. Consequently, ICA, which extracts statistically independent vectors related to the “source” signals, and CCSWA, which determines a common space describing the dispersion of all the data, were applied to the data set in order to obtain signals that were chemically easier to interpret and in order to study the effect of drying methods, time, and region of harvest on EO chemical composition.

#### 3.4.3. Independent Component Analysis (ICA)

Independent component analysis (ICA) is a technique for blind source separation (BSS) [47,48]. It aims to recover underlying “source” signals from a set of signals where they are mixed in unknown proportions, based on the assumption that these source signals are statistically independent [49]. ICA retrieves ICs and maximizes the statistical independence among the extracted source signals by using a linear transformation that maximizes the non-Gaussianity of these signals [50,51]. Therefore, ICA can reveal the physically meaningful signal sources, whereas the latent vectors calculated by PCA may represent combinations of different phenomena, and, most of the time, cannot describe a physical reality [52].

The general ICA model is given by:

X = A × S
(1)

where X is the matrix of observed signals, S is the matrix of unknown source signals (the ICs), and A is the mixing matrix of unknown coefficients directly related to the corresponding proportions of the signals (contributions of source signals to the observed signals).

The observed signals (peak areas of the chromatograms in the rows of X) are considered to be linear mixtures of source signals (rows of S) weighted by the corresponding values of A.

ICA aims to determine both A (proportions) and S (ICs), knowing only X, so it calculates a demixing matrix, W, that approximates the inverse mixing matrix so that the source signals (rows of the matrix S) may be recovered from the matrix of the measured signals (matrix X) by [53,54]:

S = W × X
(2)

In this paper, the Joint Approximate Diagonalization of Eigen matrices (JADE) algorithm was used to calculate W [51]. JADE aims to extract independent non-Gaussian sources from signal mixtures with Gaussian noise. It is based on the construction of a fourth order cumulants tensor array from the data [51].

In this paper, each row of the data matrix X, termed “observation,” corresponds to a sample signal (the peak areas of the chromatograms), which represents a mixture of source signals in unknown proportions. The columns of X are termed “variables,” and represent the chemical compounds identified.

The choice of the number of independent components is a very crucial step to consider in an ICA data treatment. In our case, ICA-DA, an extension of ICA inspired by the work of Gustafsson, was introduced in order to make a pertinent choice [55]. This method orients the extraction of signals towards those that favor the discrimination of predefined groups of samples. It consists of creating a new matrix, Z, made by the concatenation of 2 matrices, the original matrix X of the peak areas of the chromatograms (observed signals) and the matrix Y, corresponding to the information about the groups of samples. The matrix Y contains vectors corresponding to the predefined groups and highlighting the studied factors: geographical location of harvest (two sites implying two predefined groups), time of harvest (twelve months implying twelve predefined groups), and drying methods (two methods implying two predefined groups). The matrix Y contains as many rows as there are individuals in the matrix X, and as many columns as there are predefined groups. All values in this matrix are zeros, except when an individual is a member of a group, in which case the cell contains a 1. This matrix of predefined groups is then scaled by multiplying all values by the maximum value in the X matrix. Subsequently, ICA is applied to this new matrix Z (X, Y). The number of ICs, which gives a maximum discrimination of samples is chosen [11,56]. The source signals contain contributions from both the MS variables and from the scaled groups matrix. The corresponding proportions are influenced by the predefined groups. It is therefore necessary to recalculate the proportions using only the original matrix X, in order to determine whether the MS variables selected by ICA-DA are able alone to discriminate the samples into the predefined groups.

#### 3.4.4. Common Components and Specific Weight Analysis Method (CCSWA)

CCSWA is a method designed to simultaneously analyze several different data tables describing the same samples. CCSWA estimates the dispersion of the samples in a series of dimensions that are common to all the data tables. Each table has a specific weight or contribution (called salience) to the definition of each dimension of this common space [54,57,58].

Significant differences in the values of saliences for a given dimension reflect the fact that the dimension contains different amounts of information from each block [59].

Gas chromatography coupled to mass spectrometry generates a large amount of data for each analyzed sample. In this paper, the tables considered for the CCSWA method are in fact the different chromatographic peak surfaces of the EO chemical compounds for the oil samples. The data therefore consisted of 54 tables corresponding to the 54 compounds found in the GC-MS signals for each of the 94 samples. CCSWA determines a common space describing the dispersion of all the data sets; each table having a specific weight (or “salience”) associated with each dimension in this common space.

## 4. Conclusions

In conclusion, the aim of this study was to evaluate the effect of the drying methods, time, and region of harvest on the yield and the chemical composition of *O. ehrenbergii* EO. The EO analysis of *O. ehrenbergii* extracted by hydrodistillation from plant material harvested in Aabadiye and Qartaba highlighted the presence of six major constituents, with a predominance of carvacrol. This led us to the conclusion that the studied populations belong to the carvacrol chemotype. In addition to carvacrol, the most abundant compounds present in the EOs were *p*-cymene, *γ*-terpinene, thymol methyl oxide, thymoquinone, and *β*-bisabolene.

Our study showed that different drying techniques (lyophilization and shade-drying at 4 °C) did not affect the composition and yield of EOs. Location, soil characteristics, and geographical factors did not affect the chemical composition of *O. ehrenbergii.* The main factor influencing EO yield and composition was the harvest time, which affected the relative presence of carvacrol, *p*-cymene, *γ*-terpinne, *β*-bisabolene, thymol methyl oxide, and thymoquinone. What was remarkable in our study is that the flowering period (June and July) affected neither the relative proportion of carvacrol, nor the overall composition of the EO. A harvesting period from March until September would give a maximal antimicrobial property (correlated with carvacrol content), with the highest yields obtainable from June to July. Harvesting *O. ehrenbergii* in Aabadiye in January and February would yield a thymoquinone-rich EO with higher anti-inflammatory and cytotoxic properties.

## Figures and Tables

**Table 1:** Characteristics of the two regions of harvest and the chemical parameters of the soils.

	Qartaba	Aabadiye
Altitude (m)	1250	600
pH	6.53	6.28
Organic Matter (%)	4.11	4.55
P2O5 (mg/kg)	19.1	8.33
K2O (mg/kg)	234.32	53.07
N (%)	0.345	0.248

## Supplementary Materials

Supplementary Materials can be found at https://www.mdpi.com/1422-0067/20/5/1026/s1.

## Abbreviations

EO	Essential oil
PCA	Principal component analysis
ICA	Independent component analysis
CCSWA	Common component and specific weight analysis
