# Supplementary material for: Chemical Variability of the Essential Oil of Origanum ehrenbergii Boiss. from Lebanon, Assessed by Independent Component Analysis (ICA) and Common Component and Specific Weight Analysis (CCSWA)
Source: Int J Mol Sci. 2019 Feb 27;20(5):1026. doi: 10.3390/ijms20051026 (PMC6429486; doi:10.3390/ijms20051026)
Supplement: Supplementary file 1 [file ijms-20-01026-s001.pdf]

International Journal of
Molecular Sciences

MDPI

Article

Chemical Variability of the Essential Oil of *Origanum ehrenbergii* Boiss. from Lebanon, Assessed by Independent Component Analysis (ICA) and Common Component and Specific Weight Analysis (CCSWA)

Raviella Zgheib [1,2], Marc El-Beyrouthy [2,*], Sylvain Chaillou [1], Naim Ouaini [2], Douglas N. Rutledge [3], Didier Stien [4], Amine Kassouf [5], Marco Leonti [6] and Marcello Iriti [7,*]

1 Institut Jean-Pierre Bourgin, AgroParisTech, INRA, Université Paris-Saclay, RD 10, Route de Saint-Cyr, 78026 Versailles, France, raviella-zgheib@hotmail.com (R.Z.), sylvain.chaillou1@gmail.com (S.C.)
2 Holy Spirit University of Kaslik, B.P. 446 Jounieh, Lebanon, naimouaini@usek.edu.lb
3 UMR Ingénierie Procédés Aliments, AgroParisTech, INRA, Université Paris-Saclay, F-91300 Massy, France, rutledge@agroparistech.fr
4 Laboratoire de Biodiversité et Biotechnologies Microbiennes (LBBM), Observatoire Océanologique, Sorbonne Universités, UPMC Univ Paris 06, CNRS, 66650 Banyuls-sur-mer, France, didier.stien@cnrs.fr
5 Department of Chemistry and Biochemistry, Faculty of Sciences II, Lebanese University, 90656 Jdeideth El Matn, Lebanon; aminekassouf@hotmail.com
6 Department of Biomedical Sciences, University of Cagliari, Via Ospedale 72, 09124 Cagliari, Italy, marcoleonti@aim.com
7 Department of Agricultural and Enviromental Sciences, Milan State University, via G. Celoria 2, 20133 Milan, Italy
* Correspondence: marcelbeyrouthy@usek.edu.lb (M.E.-B.), marcello.iriti@unimi.it (M.I.); Tel.: 009619220884 (M.E.-B.), 0390250316766 (M.I.); Fax: 009619 6008871

Received: 18 January 2019; Accepted: 21 February 2019; Published: date

Supplementary materials

Table S1. The geographical distribution of *O. ehrenbergii* in Lebanon [4].

Region	District	Governorate
Hrajel	Keserwan	Mount Lebanon
Faraya	Keserwan	Mount Lebanon
Mayrouba	Keserwan	Mount Lebanon
Ain Al Qabou	Matn	Mount Lebanon
Zaarour	Matn	Mount Lebanon
Sannine	Matn	Mount Lebanon
Beit Méri	Matn	Mount Lebanon
Broummana	Matn	Mount Lebanon
Choueir	Matn	Mount Lebanon
Dhour Choueir	Matn	Mount Lebanon
Bikfaya	Matn	Mount Lebanon
Beit Chebab	Matn	Mount Lebanon
Aabadiye	Baabda	Mount Lebanon
Salima	Baabda	Mount Lebanon
Falougha	Baabda	Mount Lebanon
Qartaba	Jbeil	Mount Lebanon
Aïn Zehalta	Chouf	Mount Lebanon
Jabal Barouk	Chouf	Mount Lebanon
Jabal Kneissé	Chouf	Mount Lebanon
Zahlé	Zahlé	Beqaa
Jeba'a	Nabatiyeh	Nabatiyeh

Table S2. Chemical composition of the essential oils of *O. ehrenbergii* harvested from Qartaba.

				**Date of harvest**	**Apr-13**			**May-13**				**Jun-13**				**Jul-13**			
				**Drying method**	**L1**	**L2**	**D2**	**L1**	**L2**	**D1**	**D2**	**L1**	**L2**	**D1**	**D2**	**L1**	**L2**	**D1**	**D2**
				**Yield (%)**	2.76	2.6	0.9	2.8	2.9	2	1.9	3.37	3.8	3.05	2.93	3.66	4.01	2.99	3
**Ri**[a]	**Ri**[b]	**Ri**[c]	**Ri**[d]	**Chemical compounds**															
924	931	1035	1035	**α- thujene**	0.2	0.3	0.3	0.3	0.4	0.3	0.6	0.6	0.8	0.4	0.7	0.8	1.0	0.8	0.8
933	939	1076	1076	**α-pinene**	0.1	0.2	0.2	0.2	0.3	0.2	0.4	0.4	0.4	0.2	0.4	0.4	0.5	0.5	0.4
944	957			**thuja-2,4-(10)-diene**															
947	953	1076	1076	**camphene**				t	t			t	t			t	t	t	t
973	976	1132	1132	**sabinene**												t			
975	978	1312	1312	**1-octen-3-ol**		0.1	0.1	t	t			0.1	0.1	t		0.1	0.1	0.1	0.1
980	980	1118	1118	**β-pinene**		t	t	t	0.1	t	t	t	0.1	t	t	0.1	0.1	t	t
986	989	1251	1251	**3-octanone**	0.4	0.2	0.2	0.2	0.2	0.1	0.2	0.1	0.3	0.1	0.3	0.1	0.2	0.1	0.1
991	991	1174	1174	**myrcene**	0.2	0.2	0.2	0.3	0.4	0.2	0.4	0.5	0.7	0.3	0.5	0.6	0.7	0.5	0.4
996				**3-octanol**	0.3	0.5	0.5	0.3	0.4	0.4	0.4	0.4	0.2	0.4	0.3	0.9	0.4	1.0	0.5
1003	1005	1188	1188	**α-phellandrene**		0.1	0.1	0.1	0.1	0.1	0.1	0.1	0.1	0.1	0.1	0.2	0.1	0.2	0.1
1009	1011	1157	1159	**δ-3-carene**					t			t	t			0.1	t	0.1	t
1014	1018	1188	1189	**α-terpinene**	0.2	0.2	0.2	0.3	0.1	0.2	0.3	0.5	0.3	0.2	0.4	0.5	0.4	0.6	0.4
1024	1026	1280	1278	***p*-cymene**	1.8	1.7	2.9	1.6	2.3	2.8	1.9	1.6	2.1	3.4	3.3	2.4	3.1	4.4	4.7
1027	1031	1218	1218	**β-phellandrene**					0.3		0.3	0.3	0.3		0.4	0.3	0.3	0.4	0.3
1027	1032	1213	1213	**1,8-cineole**	0.1														
1043	1040	1269	1269	**(*Z*)-β-ocimene**								t	t			t	t		
1057	1062	1255	1256	**γ-terpinene**	1.4	1.3	0.6	2.2	1.3	0.8	1.3	2.9	1.6	1.1	0.8	3.4	1.9	1.9	0.7
1063	1097	1556	1556	***cis*-sabinene hydrate**	0.2		t	t		t		t	t	0.1		t	0.1	t	0.2
1086	1088	1265	1265	**α-terpinolene**												t	t	t	t
1089		1450	1452	***p*-cymenene**															
1098	1098	1553	1553	**linalool**													t		t
1160	1165	1719	1719	**borneol**															
1174	1177	1611	1611	**terpinen-4-ol**		t		t		t						t	t	t	t
1201	1189	1706	1706	**α-terpineol**															
1217	1200	1611	1602	***trans*-dihydrocarvone**															t
1238	1235	1607	1609	**thymol methyl oxide**	2.6	2.2	2.6	3.6	2.0	4.6	2.3	2.5	3.0	2.7	3.6	3.9	2.6	4.1	2.5
1249	1249	1701		**thymoquinone**														t	t
1294	1290	2198	2198	**thymol**													t		t
1300	1298	2239	2239	**carvacrol**	87.0	89.7	86.6	86.4	89.5	83.5	86.7	86.1	86.3	86.4	85.0	82.6	85.5	81.9	85.3
1350	1351	1466	1466	**α-cubebene**															
1378	1376	1497	1497	**α-copaene**	0.1	0.1	0.1	0.1	0.1	0.1	0.1	0.1	0.1	0.1	0.1	0.1	0.1	0.1	t
1386	1384	1535	1535	**β-bourbonene**												t	t		t

1416	1418	1612	1612	***β*-caryophyllene**	0.3	0.4	0.4	0.6	0.1	0.5	0.5	0.6	0.5	0.2	0.6	0.6	0.8	0.6	0.9
1436		1573	1573	***trans*-*α*-bergamotene**	0.1	t	0.1	0.1	t	0.1	t	0.1	t	t	0.1	t	t	t	t
1438	1439	1628	1628	**aromadendrene**															
1454	1454	1668	1670	***α*-humulene**	0.1	0.1	0.1	0.1	t	0.1	0.1	0.1	0.1	t	0.1	0.1	0.1	0.1	0.1
1457	1458	1689	1689	***trans*-*β*-farnesene**								t				t			t
1478	1477	1704	1704	***γ*-muurolene**		t	t	t		t	t	t	t	t		t	t	t	t
1480	1480	1726	1726	**germacrene D**		t		t				t	t			t	t	t	t
1492		1708	1707	**ledene**		t	t	t			t		t	t	t			t	t
1497		1756	1756	**bicyclogermacrene**								0.1				t	t		
1500		1740	1740	***α*-muurolene**						t									
1507	1509	1741	1743	***β*-bisabolene**	4.2	2.2	3.1	2.8	1.5	3.9	2.5	2.4	2.2	2.1	2.5	2.1	1.0	1.9	0.8
1513	1513	1776	1776	***γ*-cadinene**							t	t						t	t
1526	1524	1773	1773	***δ*-cadinene**	0.3	0.2	0.2	0.2	0.1	0.3	0.2	0.2	0.2	0.2	0.2	0.1	0.1	0.1	0.1
1540		1784	1784	***trans*-*α*-bisabolene**	0.1	0.1	0.1	0.1	0.0	0.1	0.1	0.1	0.1	t	0.1	0.1	t	t	t
1558		1984	1984	***γ*-calacorene**															t
1577	1576	2152	2150	**spathulenol**	0.1		t	0.1	0.1	0.1	t		0.1	0.1	0.1		0.1		0.1
1581	1581	2008	2008	**caryophyllene oxide**	0.2	0.1	0.2	0.1	0.3	0.3	0.1	t	0.1	0.3	0.1	0.1	0.1	0.1	0.2
1608	1606	2202	2071	**humulene-1,2-epoxide**													t		t
1640		2316	2316	***β*-caryophylladienol**															t
1686	1683	2229	2229	***α*-bisabolol**			t	t		t				t					t
2115	1949	2603	2622	**phytol**															
				**Monoterpene hydrocarbons**	3.9	3.9	4.3	4.9	5.2	4.6	5.3	6.8	6.4	5.7	6.5	8.7	8.1	9.3	7.8
				**Oxygenated monoterpenes**	89.9	91.9	89.3	90.0	91.6	88.0	89.1	88.6	89.3	89.2	88.7	86.6	88.2	86.0	88.1
				**Sesquiterpene hydrocarbons**	5.1	2.9	4.1	3.9	1.8	5.0	3.4	3.6	3.1	2.7	3.7	3.1	2.2	2.8	1.9
				**Oxygenated sesquiterpenes**	0.2	0.1	0.2	0.2	0.3	0.4	0.1	0.0	0.2	0.4	0.1	0.1	0.2	0.1	0.3
				**Others**	0.7	0.8	0.8	0.5	0.5	0.5	0.6	0.5	0.6	0.4	0.6	1.1	0.7	1.1	0.7
				**Total identified**	**102.6**	**99.6**	**98.7**	**99.5**	**99.4**	**98.6**	**98.4**	**102.9**	**99.6**	**98.4**	**99.6**	**99.6**	**99.3**	**102.4**	**98.8**

				**Date of harvest**	**Aug-13**				**Sep-13**				**Oct-13**		**Nov-13**		**Dec-13**	
				**Drying method**	**L1**	**L2**	**D1**	**D2**	**L1**	**L2**	**D1**	**D2**	**L1**	**D1**	**L1**	**D1**	**L1**	**D1**
				**Yield (%)**	2.64	2.13	2.41	1.7	2.01	1.9	1.4	1.7	1.50	1.5	1.3	1.2	0.8	1.14
**Ri[a]**	**Ri[b]**	**Ri[c]**	**Ri[d]**	**Chemical compounds**														
924	931	1035	1035	***α*- thujene**	0.8	0.9	0.8	0.9	0.5	0.5	0.5	0.3	0.4	0.2	0.4	0.3	0.5	0.1
933	939	1076	1076	***α*-pinene**	0.5	0.6	0.5	0.5	0.4	0.4	0.4	0.2	0.4	0.2	0.4	0.3	0.4	0.1
944	957			**thuja-2,4-(10)-diene**	t	t	t	t	t	0.1	0.1	0.1	0.1	0.1	0.1	0.1	0.1	t
947	953	1076	1076	**camphene**	t	0.1	t	0.1	0.1	0.1	0.1	0.1	0.1	t	0.1		0.1	
973	976	1132	1132	**sabinene**													0.1	
975	978	1312	1312	**1-octen-3-ol**	0.1	t	0.1	t	0.1	0.1	0.1	0.1	0.1	0.1	0.1	0.1	0.2	0.1

980	980	1118	1118	***β*-pinene**	0.1	0.1	0.1	0.1	t	t	0.1	t	t	t	t			
986	989	1251	1251	**3-octanone**	0.1	0.3	0.1	0.4	0.4	0.5	0.4	0.3	0.7	0.6	0.7	0.7	1.2	1.1
991	991	1174	1174	**myrcene**	0.4	0.3	0.3										0.3	
996				**3-octanol**	0.5	0.2	0.5	0.2	0.4	0.5	0.5	0.8	1.0	0.9	0.5	0.5	1.6	1.2
1003	1005	1188	1188	***α*-phellandrene**	0.1	0.1	0.1		t									
1009	1011	1157	1159	***δ*-3-carene**	t	t	t	t	t	t	t		t		t			
1014	1018	1188	1189	***α*-terpinene**	0.2	0.1	0.2	0.1	0.1	0.1	0.1	0.1	0.1	0.1	0.1	0.1	0.3	0.1
1024	1026	1280	1278	***p*-cymene**	4.1	4.9	4.8	5.1	5.5	7.3	5.3	6.1	5.7	4.9	7.2	9.2	9.7	6.7
1027	1031	1218	1218	***β*-phellandrene**	0.3		0.3						0.3					
1027	1032	1213	1213	**1,8-cineole**													0.2	
1043	1040	1269	1269	**(*Z*)-*β*-ocimene**														
1057	1062	1255	1256	***γ*-terpinene**	0.5	0.1	0.2										0.5	
1063	1097	1556	1556	***cis*-sabinene hydrate**	0.1	t	0.1	0.1	0.1	0.3	0.2	0.2	0.2	0.5	0.2	0.3	0.2	0.6
1086	1088	1265	1265	***α*-terpinolene**	t		t		t									
1089		1450	1452	***p*-cymenene**						t		t	t		0.1		0.1	
1098	1098	1553	1553	**linalool**					t		t	t	t	t	0.1			0.1
1160	1165	1719	1719	**borneol**						t		t			t		t	
1174	1177	1611	1611	**terpinen-4-ol**	t	0.1	t	0.1	0.1	0.2	0.1	0.2	0.1	0.4	0.2	0.4	0.4	0.4
1201	1189	1706	1706	***α*-terpineol**														
1217	1200	1611	1602	***trans*-dihydrocarvone**			0.1	0.1	0.1				0.1					
1238	1235	1607	1609	**thymol methyl oxide**	3.0	4.7	3.0	4.8	4.8	3.5	5.1	3.5	3.5	3.5	4.7	5.0	4.5	3.6
1249	1249	1701		**thymoquinone**	0.1		0.2	0.4	0.7	0.9	1.6	2.3	0.6	2.9	0.7	1.2	0.3	1.3
1294	1290	2198	2198	**thymol**			t		t	t			t		t			
1300	1298	2239	2239	**carvacrol**	86.1	84.1	84.9	79.1	81.9	81.0	81.0	79.4	82.4	78.8	78.9	76.8	76.3	75.8
1350	1351	1466	1466	***α*-cubebene**														
1378	1376	1497	1497	***α*-copaene**	t	t	t	t	t		t	t	t	t	t		t	
1386	1384	1535	1535	***β*-bourbonene**														
1416	1418	1612	1612	***β*-caryophyllene**	0.6	0.5	0.7	0.5	0.4	0.2	0.4	0.3	0.2	0.2	0.2	0.2	0.2	0.3
1436		1573	1573	***trans*-*α*-bergamotene**	t	t	t	t	t	0.1	t	0.1	t	t	0.1	0.1	t	t
1438	1439	1628	1628	**aromadendrene**														
1454	1454	1668	1670	***α*-humulene**	0.1	0.1	0.1	0.1	0.1	0.1	0.1	0.1	0.1	0.1	0.1	0.1	0.1	0.1
1457	1458	1689	1689	***trans*-*β*-farnesene**														
1478	1477	1704	1704	***γ*-muurolene**			t											
1480	1480	1726	1726	**germacrene D**														
1492		1708	1707	**ledene**			t											
1497		1756	1756	**bicyclogermacrene**														
1500		1740	1740	***α*-muurolene**														
1507	1509	1741	1743	***β*-bisabolene**	0.9	0.9	0.9	0.9	0.9	0.8	1.0	0.8	0.6	0.9	0.9	1.0	1.0	1.3
1513	1513	1776	1776	***γ*-cadinene**											t		t	
1526	1524	1773	1773	***δ*-cadinene**	0.1	t	0.1	t	t	t	t	t	t	t	t	0.1	t	0.1
1540		1784	1784	***trans*-*α*-bisabolene**	t	t	t		t	t					t		t	

1558		1984	1984	**γ-calacorene**	0.1	0.1	0.1	0.1	0.1	0.2	0.1	0.3	0.1	0.2	0.2	0.1	0.1	0.1
1577	1576	2152	2150	**spathulenol**	t	t	t	t	0.1	0.0	0.1	0.1	0.1	0.1	0.1	0.1		t
1581	1581	2008	2008	**caryophyllene oxide**	0.3	0.4	0.3	0.4	0.6	0.7	0.7	1.0	0.7	0.9	0.7	1.1	0.6	1.0
1608	1606	2202	2071	**humulene-1,2-epoxide**	0.1		0.1	0.1	0.1	0.1	0.1	0.1	0.1		0.1	0.1	0.1	0.2
1640		2316	2316	**β-caryophylladienol**												0.1		
1686	1683	2229	2229	**α-bisabolol**											t			
2115	1949	2603	2622	**phytol**														
				**Monoterpene hydrocarbons**	6.9	7.1	7.3	6.7	6.6	8.6	6.5	6.8	7.0	5.5	8.2	10.0	11.9	7.1
				**Oxygenated monoterpenes**	89.1	88.9	88.1	84.2	87.0	85.0	86.4	83.3	86.4	83.2	84.1	82.5	81.6	80.5
				**Sesquiterpene hydrocarbons**	1.7	1.6	1.8	1.5	1.4	1.4	1.5	1.6	0.9	1.3	1.4	1.6	1.4	1.8
				**Oxygenated sesquiterpenes**	0.4	0.4	0.4	0.5	0.7	0.8	0.8	1.2	0.9	0.9	0.9	1.4	0.7	1.2
				**Others**	0.8	0.5	0.9	1.1	1.5	2.0	2.6	3.5	2.4	4.6	2.0	2.6	3.2	3.7
				**Total identified**	**98.9**	**98.4**	**98.5**	**95.7**	**97.3**	**97.7**	**97.9**	**98.1**	**97.7**	**95.6**	**96.7**	**98.1**	**99.6**	**94.3**

				**Date of harvest**	**Jan-14**	**Feb-14**	**Mar-14**	**Apr-14**		**May-14**		**Jun-14**	
				**Drying method**	**L1**	**L1**	**L1**	**L1**	**D1**	**L1**	**D1**	**L1**	**D1**
				**Yield (%)**	0.86	1.16	1.14	2.26	0.87	2.39	1.81	2.5	1.9
**Ri[a]**	**Ri[b]**	**Ri[c]**	**Ri[d]**	**Chemical compounds**									
924	931	1035	1035	**α- thujene**	0.2		0.3	0.4	0.8	0.5	0.8	0.9	1.4
933	939	1076	1076	**α-pinene**	0.2	0.2	0.2	0.2	0.6	0.3	0.5	0.5	0.8
944	957			**thuja-2,4-(10)-diene**	t								
947	953	1076	1076	**camphene**			t	t				t	
973	976	1132	1132	**sabinene**						t		0.1	
975	978	1312	1312	**1-octen-3-ol**	0.3	0.3	0.1	0.1	0.1	t	0.1	t	0.1
980	980	1118	1118	**β-pinene**			t	t	0.1		0.1		0.1
986	989	1251	1251	**3-octanone**	1.0	0.3	0.3	0.2	0.3	0.3	0.7	0.2	0.6
991	991	1174	1174	**myrcene**			0.3	0.4	0.9	0.5	0.9	0.6	1.1
996				**3-octanol**	1.5	1.9	0.9	0.3	0.7	0.2	0.4	0.4	0.7
1003	1005	1188	1188	**α-phellandrene**			0.1	0.1	0.4	0.1	0.9	0.1	0.8
1009	1011	1157	1159	**δ-3-carene**				t			0.2		
1014	1018	1188	1189	**α-terpinene**	0.1	0.1	0.3	0.3	0.6	0.4	0.6	0.4	0.6
1024	1026	1280	1278	***p*-cymene**	4.8	3.3	3.5	1.7	3.7	1.3	4.3	2.2	4.5
1027	1031	1218	1218	**β-phellandrene**				0.2	0.5	0.2			
1027	1032	1213	1213	**1,8-cineole**	0.2	1.2							
1043	1040	1269	1269	**(Z)-β-ocimene**				t				t	
1057	1062	1255	1256	**γ-terpinene**		0.3	2.3	1.8	2.7	2.6	2.5	2.1	2.2
1063	1097	1556	1556	***cis*-sabinene hydrate**	0.2	0.3	0.1	t		0.1		t	
1086	1088	1265	1265	**α-terpinolene**				t				t	

1089		1450	1452	***p*-cymenene**			0.1						
1098	1098	1553	1553	**linalool**							0.1		
1160	1165	1719	1719	**borneol**			t				0.3		0.2
1174	1177	1611	1611	**terpinen-4-ol**	0.1		t	t			0.1	t	
1201	1189	1706	1706	***α*-terpineol**									
1217	1200	1611	1602	***trans*-dihydrocarvone**									
1238	1235	1607	1609	**thymol methyl oxide**	3.9	3.9	2.6	1.9	3.0	3.2	4.3	3.0	4.7
1249	1249	1701		**thymoquinone**	1.1	0.6							
1294	1290	2198	2198	**thymol**							t		
1300	1298	2239	2239	**carvacrol**	76.5	58.5	82.8	87.6	77.9	85.0	74.8	85.3	74.5
1350	1351	1466	1466	***α*-cubebene**					0.1		0.3	t	0.2
1378	1376	1497	1497	***α*-copaene**		0.2	0.1	0.1	0.2	0.1	0.1	0.1	0.2
1386	1384	1535	1535	***β*-bourbonene**				t					
1416	1418	1612	1612	***β*-caryophyllene**	0.2	0.7	0.7	0.5	0.9	0.9	0.9	0.9	1.3
1436		1573	1573	***trans*-*α*-bergamotene**	t	0.1	0.1	0.1	0.1	0.1	0.1	0.1	0.1
1438	1439	1628	1628	**aromadendrene**									
1454	1454	1668	1670	***α*-humulene**	0.1	0.2	0.1	0.1	0.2	0.1	0.2	0.2	0.2
1457	1458	1689	1689	***trans*-*β*-Farnesene**		0.1		t	0.1	t		t	0.1
1478	1477	1704	1704	***γ*-muurolene**			t	t	0.1	t	t	t	t
1480	1480	1726	1726	**germacrene D**		0.1	t	t	t	t	t	t	t
1492		1708	1707	**ledene**			0.1	t	0.1	0.1	0.1		
1497		1756	1756	**bicyclogermacrene**								0.1	0.1
1500		1740	1740	***α*-muurolene**			t						
1507	1509	1741	1743	***β*-bisabolene**	2.5	5.0	3.5	2.7	4.3	2.8	3.4	2.0	2.8
1513	1513	1776	1776	***γ*-cadinene**	0.1		0.1			0.1	0.5		
1526	1524	1773	1773	***δ*-cadinene**	0.1	0.3	0.3	0.2	0.3	0.2	0.3	0.2	0.3
1540		1784	1784	***trans*-*α*-bisabolene**		0.1	0.1	0.1	0.1	0.1	0.1	0.1	0.1
1558		1984	1984	***γ*-calacorene**	0.2	0.1							
1577	1576	2152	2150	**spathulenol**	t	0.2	0.1	0.1	0.1	t	0.1	t	0.1
1581	1581	2008	2008	**caryophyllene oxide**	0.9	1.4	0.2	0.1		t	0.1	0.1	0.1
1608	1606	2202	2071	**humulene-1,2-epoxide**	0.1	0.4	t						
1640		2316	2316	***β*-caryophylladienol**		0.1		t					
1686	1683	2229	2229	***α*-bisabolol**			t	t					
2115	1949	2603	2622	**phytol**		0.4		0.0					
				**Monoterpene hydrocarbons**	5.2	3.9	7.1	5.1	10.1	5.9	10.8	6.9	11.5
				**Oxygenated monoterpenes**	81.0	63.9	85.5	89.5	81.0	88.3	79.6	88.4	79.4
				**Sesquiterpene hydrocarbons**	3.1	6.9	4.9	3.7	6.4	4.5	5.9	3.6	5.3
				**Oxygenated sesquiterpenes**	1.0	2.2	0.3	0.2	0.1	0.0	0.2	0.1	0.3
				**Others**	3.9	3.5	1.2	0.6	1.1	0.5	1.2	0.7	1.4
				**Total identified**	**94.2**	**80.4**	**99.0**	**99.1**	**98.7**	**99.2**	**97.7**	**99.6**	**97.8**

				**Date of harvest**	**Jul-14**		**Aug-14**		**Sep-14**		**Oct-14**		**Nov-14**	**Dec-14**	
				**Drying method**	**L1**	**D1**	**L1**	**D1**	**L1**	**D1**	**L1**	**D1**	**L1**	**L1**	**D1**
				**Yield (%)**	4.04	3.47	3.46	3.5	3.02	1.8	1.87	1.42	0.77	0.8	0.75
**Ri[a]**	**Ri[b]**	**Ri[c]**	**Ri[d]**	**Chemical compounds**											
924	931	1035	1035	**α- thujene**	1.0	0.9	0.7	0.9	0.7	0.7	0.4	0.2	0.2	0.7	0.3
933	939	1076	1076	**α-pinene**	0.5	0.5	0.4	0.6	0.5	0.5	0.3	0.1	0.5	0.6	0.3
944	957			**thuja-2,4-(10)-diene**			t	t	t	t		t	0.1	t	
947	953	1076	1076	**camphene**	t	t	t	0.1	0.1	0.1	0.1		0.2	0.1	
973	976	1132	1132	**sabinene**	t	t	t	t						t	
975	978	1312	1312	**1-octen-3-ol**	t		0.1	0.1	0.3	0.3	0.2	0.1	0.2	0.2	0.2
980	980	1118	1118	**β-pinene**	0.1	t	0.1	0.1	0.1	0.1	0.1			0.1	
986	989	1251	1251	**3-octanone**	0.1	0.2	0.1	0.1	0.3	0.4	1.0	0.9	1.5	0.9	1.2
991	991	1174	1174	**myrcene**	0.6	0.5	0.3	0.2	0.2					0.8	0.4
996				**3-octanol**	0.2	0.2	0.7	0.8	0.9	0.9	1.5	1.5	1.8	1.9	2.1
1003	1005	1188	1188	**α-phellandrene**	0.1	0.1	0.1	0.1	0.1	0.1	0.9	0.6		0.4	1.5
1009	1011	1157	1159	**δ-3-carene**	t		t	t	t	t					
1014	1018	1188	1189	**α-terpinene**	0.3	0.2	0.2	0.3	0.3	0.2	0.3	0.2	0.3	0.8	0.6
1024	1026	1280	1278	***p*-cymene**	2.3	3.0	5.2	7.5	8.7	10.6	8.8	6.8	16.6	12.6	14.6
1027	1031	1218	1218	**β-phellandrene**	0.3	0.3	0.4	0.4		0.4					
1027	1032	1213	1213	**1,8-cineole**											
1043	1040	1269	1269	**(Z)-β-ocimene**	t									t	
1057	1062	1255	1256	**γ-terpinene**	1.1	0.6	0.4	0.1	0.4				0.3	4.8	1.4
1063	1097	1556	1556	***cis*-sabinene hydrate**	t	0.1	0.1	0.2	t	0.3	0.3	0.5	0.4	0.2	0.4
1086	1088	1265	1265	**α-terpinolene**	t		0.1	0.1	0.1						
1089		1450	1452	***p*-cymenene**						0.1	0.1		0.2	0.2	
1098	1098	1553	1553	**linalool**			t	t	t	t				0.1	
1160	1165	1719	1719	**borneol**						t	0.3	0.2		0.1	0.5
1174	1177	1611	1611	**terpinen-4-ol**	t		0.1	0.1	0.2	0.2	0.5	0.3	0.4	0.4	
1201	1189	1706	1706	**α-terpineol**				t							
1217	1200	1611	1602	***trans*-dihydrocarvone**											
1238	1235	1607	1609	**thymol methyl oxide**	3.2	3.5	3.2	3.4	1.7	2.0	4.9	5.2	9.3	7.2	9.1
1249	1249	1701		**thymoquinone**			0.2	0.8	0.4	1.8		0.1			
1294	1290	2198	2198	**thymol**	t		t	t		t	t			t	
1300	1298	2239	2239	**carvacrol**	87.1	85.7	84.1	79.5	81.5	77.1	73.9	75.8	57.6	59.4	55.3
1350	1351	1466	1466	**α-cubebene**							0.3	0.1	0.1	0.1	0.5
1378	1376	1497	1497	**α-copaene**	0.1	0.1	t	t	t	t	t		0.1	0.1	0.1
1386	1384	1535	1535	**β-bourbonene**	t									t	
1416	1418	1612	1612	**β-caryophyllene**	0.9	1.1	0.7	0.8	0.4	0.3	0.4	0.5	0.2	0.4	0.5

1436		1573	1573	***trans*-α-bergamotene**		t		t	t	t	t	t	0.1	0.1	0.1
1438	1439	1628	1628	**aromadendrene**											
1454	1454	1668	1670	**α-humulene**	0.2	0.2	0.1	0.2	0.1	0.1	0.1	0.1	0.1	0.1	0.1
1457	1458	1689	1689	***trans*-β-Farnesene**											
1478	1477	1704	1704	**γ-muurolene**	t			t						t	0.1
1480	1480	1726	1726	**germacrene D**	t									0.1	
1492		1708	1707	**ledene**		t									
1497		1756	1756	**bicyclogermacrene**	t		t								
1500		1740	1740	**α-muurolene**											
1507	1509	1741	1743	**β-bisabolene**	1.1	1.6	0.6	0.7	0.6	0.5	1.7	1.6	1.3	2.7	3.1
1513	1513	1776	1776	**γ-cadinene**		t		t	t	t					
1526	1524	1773	1773	**δ-cadinene**	0.1	0.1	t	0.1	t	t	t	0.1	0.1	0.2	0.2
1540		1784	1784	***trans*-α-bisabolene**	t	t	t	t	t	t			0.1	0.1	0.1
1558		1984	1984	**γ-calacorene**			0.1	0.2	0.1	0.1			0.1	t	
1577	1576	2152	2150	**spathulenol**	t	t	t	0.0	t	t	t	t	0.1		
1581	1581	2008	2008	**caryophyllene oxide**	0.1	0.2	0.5	0.6	0.6	0.6	0.8	0.9	2.7	1.2	1.7
1608	1606	2202	2071	**humulene-1,2-epoxide**			0.1	0.1	0.1	0.1	0.2	0.2	0.5		0.1
1640		2316	2316	**β-caryophylladienol**						t		0.1			0.1
1686	1683	2229	2229	**α-bisabolol**				t							
2115	1949	2603	2622	**phytol**											
				**Monoterpene hydrocarbons**	6.2	6.0	7.9	10.2	11.1	12.7	11.0	7.9	18.3	21.1	19.1
				**Oxygenated monoterpenes**	90.4	89.2	87.5	83.2	83.4	79.6	79.9	81.9	67.7	67.3	65.3
				**Sesquiterpene hydrocarbons**	2.3	3.1	1.5	1.8	1.2	1.1	2.4	2.3	2.0	3.8	4.7
				**Oxygenated sesquiterpenes**	0.1	0.2	0.6	0.7	0.7	0.7	0.9	1.1	3.3	1.2	1.9
				**Others**	0.3	0.4	1.2	1.9	1.9	3.4	2.7	2.7	3.5	3.0	3.4
				**Total identified**	**99.4**	**98.8**	**98.6**	**97.9**	**98.3**	**97.6**	**96.9**	**95.8**	**94.8**	**96.5**	**94.5**

International Journal of
Molecular Sciences

Notes: Drying method (L: lyophilization; D: shade-drying at 4 °C)
Harvest number (1: harvest at the beginning of the month; 2: harvest in the middle of the month)
Retention index (Ri^a: Retention index calculated on a HP-5MS column; Ri^b: Retention index on a HP-5MS column from literature [42]; Ri^c: Retention index calculated on a HP Innowax column; Ri^d: Retention index on a HP Innowax column from literature [60,61]
t: trace (less than 0.05.%)
Blanks: not detected

Table S3. Chemical composition of the essential oils of *O. ehrenbergii* harvested from Aabadiye.

				Date of harvest	March	Apr-13		May-13				Jun-13			
				**Drying method**	**L1**	**L1**	**L2**	**L1**	**L2**	**D1**	**D2**	**L1**	**L2**	**D1**	**D2**
				**Yield (%)**	2.7	2.86	2.84	3.24	3.66	2.19	2.19	4.45	4.5	2.78	3.9
**Ri[a]**	**Ri[b]**	**Ri[c]**	**Ri[d]**	**Chemical compounds**											
924	931	1035	1035	**α- thujene**	0.1	0.4	0.2	0.5	0.4	0.3	0.3	0.7	0.7	0.8	0.8
933	939	1076	1076	**α-pinene**	0.1	0.3	0.1	0.3	0.2	0.2	0.2	0.4	0.4	0.4	0.4
944	957			**thuja-2,4-(10)-diene**											
947	953	1076	1076	**camphene**		t		t	t			t	t	t	
973	976	1132	1132	**sabinene**		t		t				t	0.1		
975	978	1312	1312	**1-octen-3-ol**	t	t		t	t	t	t	0.1	t	t	t
980	980	1118	1118	**β-pinene**	0.1	0.1	t	0.1	t	t	t	0.1	t		
986	989	1251	1251	**3-octanone**	0.5	0.4	0.3	0.3	0.2	0.2	0.2	0.3	0.2	0.2	0.2
991	991	1174	1174	**myrcene**	0.1	0.6	0.3	0.7	0.5	t	0.1	0.8	0.6	0.6	0.6
996				**3-octanol**	0.3	0.2	0.1	0.3	0.1	0.3	0.1	0.2	0.1	0.1	0.1
1003	1005	1188	1188	**α-phellandrene**		0.1	t	0.2	0.1		t	0.2	0.1	0.1	0.1
1009	1011	1157	1159	**δ-3-carene**		t		t				t	t	t	t
1014	1018	1188	1189	**α-terpinene**	0.1	0.3	0.3	1.0	0.8	0.3	0.3	0.9	0.6	0.7	0.6
1024	1026	1280	1278	***p*-cymene**	2.4	3.6	2.5	3.2	3.7	8.3	7.8	1.9	1.6	3.2	3.2
1027	1031	1218	1218	**β-phellandrene**				0.5	0.3			0.4	0.2	0.3	0.4
1027	1032	1213	1213	**1,8-cineole**	0.2										
1043	1040	1269	1269	**(Z)-β-ocimene**		t		t				t	t		
1057	1062	1255	1256	**γ-terpinene**	0.3	3.7	2.6	6.3	5.4		0.3	5.8	3.7	3.3	2.6
1063	1097	1556	1556	***cis*-sabinene hydrate**	0.1	t		t				0.2	t	t	0.1
1086	1088	1265	1265	**α-terpinolene**		t		t				t	t	t	
1089		1450	1452	***p*-cymenene**											
1098	1098	1553	1553	**linalool**											
1160	1165	1719	1719	**borneol**	t										
1174	1177	1611	1611	**terpinen-4-ol**	0.1	t		0.1				t			
1201	1189	1706	1706	**α-terpineol**				t							
1217	1200	1611	1602	***trans*-dihydrocarvone**	t			t							
1238	1235	1607	1609	**thymol methyl oxide**	3.2	2.8	2.5	4.0	3.2	3.5	3.7	1.9	1.1	1.6	1.3
1249	1249	1701		**thymoquinone**	0.3			t	0.1	1.3	0.7				
1294	1290	2198	2198	**thymol**	t	0.3						0.1			
1300	1298	2239	2239	**carvacrol**	83.6	82.8	88.0	77.7	81.9	76.1	77.9	82.5	88.6	85.2	86.8
1350	1351	1466	1466	**α-cubebene**				t				t			
1378	1376	1497	1497	**α-copaene**		0.2	0.1	0.1	0.1	0.1	0.1	0.1	t	0.1	0.1
1386	1384	1535	1535	**β-bourbonene**				t							

1416	1418	1612	1612	**β-caryophyllene**	0.6	0.3	0.5	0.8	0.7	0.3	0.5	1.0	0.6	0.9	0.7
1436		1573	1573	***trans*-α-bergamotene**	0.1	t	t	0.1	t	0.1	0.1				t
1438	1439	1628	1628	**aromadendrene**											
1454	1454	1668	1670	**α-humulene**	0.1	0.1	0.1	0.1	0.1	0.1	0.1	0.2	0.1	0.2	0.1
1457	1458	1689	1689	***trans*-β-Farnesene**			t	t	t			t	t	t	
1478	1477	1704	1704	**γ-muurolene**		t	t	t	t	t	t	t	t	t	
1480	1480	1726	1726	**germacrene D**				t	t			0.1	t	t	
1492		1708	1707	**ledene**	t	t	t	0.1	0.1		t			t	t
1497		1756	1756	**bicyclogermacrene**								0.1	0.1	t	
1500		1740	1740	**α-muurolene**		t		t		t					
1507	1509	1741	1743	**β-bisabolene**	3.8	1.9	1.9	2.2	1.4	2.5	2.5	1.1	0.7	0.9	0.8
1513	1513	1776	1776	**γ-cadinene**		t	t	0.1	t	t	t	t	t	t	
1526	1524	1773	1773	**δ-cadinene**	0.2	0.2	0.2	0.2	0.1	0.2	0.2	0.2	0.1	0.1	0.1
1540		1784	1784	***trans*-α-bisabolene**	0.1	t	t	0.1	t	t	t	t	t	t	t
1558		1984	1984	**γ-calacorene**	t					0.3	0.2				
1577	1576	2152	2150	**spathulenol**	0.2	0.2	0.1	0.1	0.1	0.2	0.2	0.2	0.1	0.2	0.2
1581	1581	2008	2008	**caryophyllene oxide**	0.9	0.5	0.1	0.1	0.1	0.7	0.6	0.1	0.1	0.1	0.2
1608	1606	2202	2071	**humulene-1,2-epoxide**	0.1	0.1				0.1	0.1				
1640		2316	2316	**β-caryophylladienol**		t		t				t		t	
1686	1683	2229	2229	**α-bisabolol**	0.1			t		t	t				
2115	1949	2603	2622	**phytol**											
				**Monoterpene hydrocarbons**	3.2	9.0	5.9	12.8	11.5	9.1	9.0	11.0	8.0	9.5	8.8
				**Oxygenated monoterpenes**	87.2	85.9	90.5	81.7	85.0	79.6	81.6	84.7	89.6	86.9	88.2
				**Sesquiterpene hydrocarbons**	4.8	2.6	2.6	3.8	2.5	3.5	3.7	2.7	1.5	2.2	1.8
				**Oxygenated sesquiterpenes**	1.2	0.7	0.2	0.2	0.2	0.9	1.0	0.4	0.2	0.3	0.4
				**Others**	1.0	0.6	0.4	0.6	0.4	1.8	1.1	0.6	0.3	0.3	0.3
				**Total identified**	**97.5**	**98.8**	**99.6**	**99.0**	**99.6**	**94.9**	**96.3**	**99.3**	**99.6**	**99.2**	**99.5**

International Journal of Molecular Sciences

MDPI

				Date of harvest	Jul-13				Aug-13				Sep-13			Oct-13	Nov-13
				**Drying method**	**L1**	**L2**	**D1**	**D2**	**L1**	**L2**	**D1**	**D2**	**L1**	**L2**	**D1**	**L1**	**L1**
				**Yield (%)**	4.52	4.53	3.45	3.56	3.69	3.69	3.01	3.1	3.19	3.10	2.99	2.99	2.26
**Ri[a]**	**Ri[b]**	**Ri[c]**	**Ri[d]**	**Chemical compounds**													
924	931	1035	1035	**α- thujene**	1.0	0.7	1.1	0.4	0.8	0.8	0.9	0.8	1.0	0.9	0.7	0.6	0.6
933	939	1076	1076	**α-pinene**	0.6	0.4	0.7	0.2	0.5	0.4	0.5	0.4	0.6	0.5	0.4	0.5	0.6
944	957			**thuja-2,4-(10)-diene**													0.1
947	953	1076	1076	**camphene**	0.1		0.1		t	t	t	t	0.1	t	t	0.1	0.1
973	976	1132	1132	**sabinene**	t		t		t				t				
975	978	1312	1312	**1-octen-3-ol**	0.1	t	0.1	t	0.1	0.1	0.1	t	0.1	0.1	0.1	0.1	0.1
980	980	1118	1118	**β-pinene**	0.1		0.1	t	0.1	0.1	0.1	0.1	0.1	0.1	0.1	0.1	0.2
986	989	1251	1251	**3-octanone**	0.2	0.3	0.2	0.5	0.3	0.2	0.3	0.3	0.5	0.3	0.5	0.7	1.0
991	991	1174	1174	**myrcene**	1.0	0.4	0.9		0.8	0.6	0.8	0.6	0.8	0.6	0.5		
996				**3-octanol**	0.4	0.1	0.5	0.1	0.2	0.2	0.2	0.2	0.3	0.2	0.3	0.3	0.6
1003	1005	1188	1188	**α-phellandrene**	0.2	0.1	0.2		0.1	0.1	0.1	0.1	0.2	0.1	0.1		
1009	1011	1157	1159	**δ-3-carene**	0.1	t	0.1	t	t	t	0.1	t	0.1	t	t		
1014	1018	1188	1189	**α-terpinene**	1.3	0.3	1.2	0.1	0.5	0.5	0.4	0.4	0.6	0.5	0.4	0.2	0.2
1024	1026	1280	1278	***p*-cymene**	3.8	2.9	5.8	3.4	2.5	2.1	3.2	3.3	3.8	3.3	4.8	9.9	15.3
1027	1031	1218	1218	**β-phellandrene**	0.6	0.3	0.6		0.4	0.3	0.4	0.3	0.5	0.3		0.5	0.6
1027	1032	1213	1213	**1,8-cineole**													
1043	1040	1269	1269	**(*Z*)-β-ocimene**	0.1		t		t	t			t				
1057	1062	1255	1256	**γ-terpinene**	7.1	0.8	6.0		2.1	2.8	1.6	1.7	2.6	2.3	0.8		
1063	1097	1556	1556	***cis*-sabinene hydrate**	0.1		0.2	0.1	0.1	t	0.1	0.1	0.1	t	0.2	0.1	0.1
1086	1088	1265	1265	**α-terpinolene**	t		t		t	t		t	t		t		
1089		1450	1452	***p*-cymenene**													0.1
1098	1098	1553	1553	**linalool**	t		0.1										
1160	1165	1719	1719	**borneol**													
1174	1177	1611	1611	**terpinen-4-ol**	t		t		t	t		t	t		0.1	0.1	0.6
1201	1189	1706	1706	**α-terpineol**	0.1		0.1						t				
1217	1200	1611	1602	***trans*-dihydrocarvone**	t								0.1				
1238	1235	1607	1609	**thymol methyl oxide**	1.9	4.5	2.0	5.2	2.0	1.3	2.3	1.7	2.5	1.5	2.4	3.3	4.3
1249	1249	1701		**thymoquinone**		t	0.1	0.4				0.2	0.2	0.1	0.5	3.3	3.2
1294	1290	2198	2198	**thymol**					t				t		t		
1300	1298	2239	2239	**carvacrol**	78.1	86.6	76.8	83.0	87.1	88.6	85.9	87.9	83.5	87.4	84.3	73.5	67.8
1350	1351	1466	1466	**α-cubebene**	t					t			t				
1378	1376	1497	1497	**α-copaene**	t	t		t	t	t	t	t	0.1	t	t	0.1	0.1
1386	1384	1535	1535	**β-bourbonene**													
1416	1418	1612	1612	**β-caryophyllene**	1.4	0.7	1.1	0.5	0.7	0.5	0.6	0.4	0.6	0.5	0.5	0.4	0.5
1436		1573	1573	***trans*-α-bergamotene**		t	t	t									

1438	1439	1628	1628	**aromadendrene**	t					t	0.1	t	0.1	t	0.1	0.1	0.1
1454	1454	1668	1670	**α-humulene**	0.3	0.1	0.2	0.1	0.1	0.1	0.1	0.1	0.1	0.1	0.1	0.2	0.2
1457	1458	1689	1689	***trans*-β-Farnesene**	t		t			t	t				t		
1478	1477	1704	1704	**γ-muurolene**						t			t		t	t	
1480	1480	1726	1726	**germacrene D**	t					t			t	t			
1492		1708	1707	**ledene**			t	t	t		t	t			t	t	0.1
1497		1756	1756	**bicyclogermacrene**	0.1					0.1			0.1	0.1			
1500		1740	1740	**α-muurolene**													
1507	1509	1741	1743	**β-bisabolene**	0.9	0.7	0.8	0.9	0.5	0.5	0.5	0.4	0.6	0.5	0.6	0.7	0.8
1513	1513	1776	1776	**γ-cadinene**				t	t	t		t	t		t		
1526	1524	1773	1773	**δ-cadinene**	t	0.1	t	0.1	0.1	0.1	0.1	0.1	0.1	0.1	0.1	0.1	0.1
1540		1784	1784	***trans*-α-bisabolene**	t		t		t	t	t	t	t		t		
1558		1984	1984	**γ-calacorene**				t							0.1	0.6	0.2
1577	1576	2152	2150	**spathulenol**	0.1	0.1	0.1	0.1	0.2	0.1	0.2	0.1	0.1	0.1	0.2	0.2	0.2
1581	1581	2008	2008	**caryophyllene oxide**	0.1	0.3	0.3	0.7	0.1	0.1	0.3	0.1	0.1		0.2	0.6	1.1
1608	1606	2202	2071	**humulene-1,2-epoxide**		t	t				t		t		t		0.2
1640		2316	2316	**β-caryophylladienol**							t	t	t		t		0.1
1686	1683	2229	2229	**α-bisabolol**													
2115	1949	2603	2622	**phytol**													
				**Monoterpene hydrocarbons**	15.7	5.8	16.8	4.2	7.8	7.7	8.0	7.7	10.2	8.6	7.8	11.9	17.6
				**Oxygenated monoterpenes**	80.1	91.2	79.2	88.2	89.2	89.9	88.3	89.6	86.2	88.9	86.9	77.0	72.7
				**Sesquiterpene hydrocarbons**	2.7	1.6	2.1	1.7	1.4	1.3	1.4	0.9	1.6	1.2	1.5	2.2	2.0
				**Oxygenated sesquiterpenes**	0.2	0.3	0.3	0.8	0.3	0.1	0.5	0.3	0.3	0.1	0.3	0.8	1.6
				**Others**	0.6	0.4	0.9	0.9	0.6	0.5	0.6	0.7	1.0	0.7	1.4	4.4	4.8
				**Total identified**	**99.4**	**99.3**	**99.3**	**95.8**	**99.3**	**99.5**	**98.8**	**99.2**	**99.3**	**99.5**	**98.0**	**96.2**	**98.6**

				Date of harvest	Jan-14		Feb-14	Mar-14	Apr-14		May-14		Jun-14	
				**Drying method**	**L1**	**D1**	**L1**	**L1**	**L1**	**D1**	**L1**	**D1**	**L1**	**D1**
				**Yield (%)**	1.06	0.79	1.18	1.11	2.44	1.52	3.74	1.81	3.97	3.09
**Ri[a]**	**Ri[b]**	**Ri[c]**	**Ri[d]**	**Chemical compounds**										
924	931	1035	1035	***α*- thujene**			0.1		0.3	0.3	0.7	0.6	0.7	0.8
933	939	1076	1076	***α*-pinene**	0.1		0.2		0.3	0.2	0.4	0.4	0.4	0.4
944	957			**thuja-2,4-(10)-diene**										
947	953	1076	1076	**camphene**								t		t
973	976	1132	1132	**sabinene**								t	t	t
975	978	1312	1312	**1-octen-3-ol**							0.1	t	0.1	t
980	980	1118	1118	***β*-pinene**	0.3	0.2	0.3		0.2		0.1	0.1	0.1	0.1
986	989	1251	1251	**3-octanone**	1.8	1.3	1.9	0.7	0.9	0.4	0.4	0.3	0.5	0.4
991	991	1174	1174	**myrcene**					0.5	0.2	0.8	0.5	0.9	0.7
996				**3-octanol**	1.4	0.9	1.4	0.4	0.5	0.3	0.2	0.2	0.3	0.2
1003	1005	1188	1188	***α*-phellandrene**					0.1		0.2	0.1	0.2	0.1
1009	1011	1157	1159	***δ*-3-carene**								t	t	t
1014	1018	1188	1189	***α*-terpinene**					0.5	0.3	0.8	0.5	1.0	0.8
1024	1026	1280	1278	***p*-cymene**	10.2	9.1	12.9	2.7	3.2	3.5	1.9	4.5	2.1	2.7
1027	1031	1218	1218	***β*-phellandrene**							0.3	0.3	0.4	0.4
1027	1032	1213	1213	**1,8-cineole**										
1043	1040	1269	1269	**(Z)-*β*-ocimene**									t	t
1057	1062	1255	1256	***γ*-terpinene**					4.0	1.1	5.5	2.2	6.4	4.7
1063	1097	1556	1556	***cis*-sabinene hydrate**	0.7	0.7	0.6			0.1	t	0.1	0.1	0.1
1086	1088	1265	1265	***α*-terpinolene**								t	t	t
1089		1450	1452	***p*-cymenene**			0.1							
1098	1098	1553	1553	**linalool**	0.4		0.3							
1160	1165	1719	1719	**borneol**	0.2		0.2							
1174	1177	1611	1611	**terpinen-4-ol**	0.8	0.5	0.9		0.1					
1201	1189	1706	1706	***α*-terpineol**										
1217	1200	1611	1602	***trans*-dihydrocarvone**	0.2		0.3							
1238	1235	1607	1609	**thymol methyl oxide**	6.9	7.1	6.5	2.2	4.6	3.2	3.2	2.4	3.7	3.2
1249	1249	1701		**thymoquinone**	10.6	12.8	11.6							
1294	1290	2198	2198	**thymol**										
1300	1298	2239	2239	**carvacrol**	48.5	50.6	48.1	73.7	71.8	82.5	81.5	83.1	79.6	82.1
1350	1351	1466	1466	***α*-cubebene**			0.1		t		0.1	t	0.1	0.1
1378	1376	1497	1497	***α*-copaene**	0.1		0.1	0.1	0.2	0.2	0.2	0.2	0.2	0.1
1386	1384	1535	1535	***β*-bourbonene**								t		t

1416	1418	1612	1612	***β*-caryophyllene**	0.2	0.2	0.3	0.9	1.0	0.8	0.7	0.6	1.0	0.9
1436		1573	1573	***trans*-*α*-bergamotene**					0.1	0.1				
1438	1439	1628	1628	**aromadendrene**	0.1		0.1	0.2			0.1		0.1	0.1
1454	1454	1668	1670	***α*-humulene**	0.2	0.2	0.2	0.2	0.2	0.1	0.1	0.1	0.2	0.2
1457	1458	1689	1689	***trans*-*β*-Farnesene**							t	t		
1478	1477	1704	1704	***γ*-muurolene**							t	t	t	t
1480	1480	1726	1726	**germacrene D**					0.1		0.1	t	0.1	0.1
1492		1708	1707	**ledene**								0.1		
1497		1756	1756	**bicyclogermacrene**					0.1		0.3		0.3	0.2
1500		1740	1740	***α*-muurolene**								t		t
1507	1509	1741	1743	***β*-bisabolene**	0.9	1.2	1.2	4.3	2.6	3.3	1.5	1.6	0.8	0.7
1513	1513	1776	1776	***γ*-cadinene**					t		0.1	0.0	t	0.0
1526	1524	1773	1773	***δ*-cadinene**	0.1		0.1	0.3	0.2	0.3	0.3	0.3	0.2	0.2
1540		1784	1784	***trans*-*α*-bisabolene**				0.1	0.1	0.1	t	t	t	t
1558		1984	1984	***γ*-calacorene**	0.6	2.5	0.7	0.2		0.1				
1577	1576	2152	2150	**spathulenol**	0.5	0.4	0.4	0.4	0.2	0.3	0.1	0.4	0.2	0.2
1581	1581	2008	2008	**caryophyllene oxide**	2.5	2.7	2.9	2.1	0.4	0.6	t	0.1	0.1	0.1
1608	1606	2202	2071	**humulene-1,2-epoxide**	0.3	0.3	0.4	0.3	0.1	0.1				
1640		2316	2316	***β*-caryophylladienol**	0.1		0.1	0.1		0.1			t	t
1686	1683	2229	2229	***α*-bisabolol**								t		
2115	1949	2603	2622	**phytol**										
				**Monoterpene hydrocarbons**	10.6	9.3	13.7	2.7	8.9	5.5	10.7	9.1	12.0	10.6
				**Oxygenated monoterpenes**	57.8	58.8	57.0	75.9	76.4	85.9	84.7	85.5	83.4	85.4
				**Sesquiterpene hydrocarbons**	2.2	4.1	2.8	6.4	4.7	4.9	3.5	2.8	3.0	2.6
				**Oxygenated sesquiterpenes**	3.5	3.4	3.8	3.0	0.6	1.0	0.1	0.5	0.2	0.2
				**Others**	13.8	15.0	14.8	1.1	1.4	0.7	0.7	0.5	0.8	0.6
				**Total identified**	**87.9**	**90.7**	**92.2**	**89.2**	**92.0**	**97.9**	**99.7**	**98.4**	**99.5**	**99.5**

International Journal of Molecular Sciences

				Date of harvest	Jul-14		Aug-14		Sep-14		Oct-14		Nov-14	Dec-14	
				Drying method	L1	D1	L1	D1	L1	D1	L1	D1	L1	L1	D1
				Yield (%)	3.35	2.83	3.44	2.70	3.43	3.03	2.93	2.66	2.18	1.7	0.63
Ri[a]	Ri[b]	Ri[c]	Ri[d]	Chemical compounds											
924	931	1035	1035	*α*- thujene	0.7	0.9	0.8	1.0	0.9	0.7	1.0	1.0	0.6	0.8	0.2
933	939	1076	1076	*α*-pinene	0.4	0.5	0.5	0.6	0.6	0.4	0.9	0.9	0.7	0.9	0.2
944	957			thuja-2,4-(10)-diene							0.1	0.1	0.1	0.1	0.1
947	953	1076	1076	camphene	t						0.1	0.1	0.1	0.1	0.1
973	976	1132	1132	sabinene	t		t					0.1	0.1		
975	978	1312	1312	1-octen-3-ol	0.1	0.1	0.1	0.1	0.1	0.1	0.1			0.2	0.1
980	980	1118	1118	*β*-pinene	0.1	0.1	0.2	0.1	0.2	0.1	0.2	0.2	0.3	0.3	0.3
986	989	1251	1251	3-octanone	0.5	0.5	0.6	0.6	0.8	0.6	1.5	1.5	1.5	1.8	1.6
991	991	1174	1174	myrcene	0.7	0.8	0.8	0.8	0.7	0.2					
996				3-octanol	0.4	0.5	0.5	0.6	0.5	0.5	0.9	1.0	1.0	0.9	0.9
1003	1005	1188	1188	*α*-phellandrene	0.2	0.2	0.2	0.2	0.2	0.1	0.4	0.4	0.4	0.3	0.2
1009	1011	1157	1159	*δ*-3-carene	t		0.1	0.1			0.1	0.1			
1014	1018	1188	1189	*α*-terpinene	0.6	0.7	0.6	0.6	0.4	0.4	0.3	0.3	0.3	0.5	0.2
1024	1026	1280	1278	*p*-cymene	2.2	3.8	3.5	4.7	6.4	6.4	16.8	15.0	17.2	20.9	14.2
1027	1031	1218	1218	*β*-phellandrene	0.4	0.5	0.4	0.5	0.4	0.4					
1027	1032	1213	1213	1,8-cineole											
1043	1040	1269	1269	(*Z*)-*β*-ocimene	t										
1057	1062	1255	1256	*γ*-terpinene	3.0	2.6	2.8	2.1	1.0						
1063	1097	1556	1556	*cis*-sabinene hydrate	0.2	0.1	t	0.1		0.1	0.1	0.1	0.4	0.4	0.8
1086	1088	1265	1265	*α*-terpinolene	t			t							0.1
1089		1450	1452	*p*-cymenene							0.1	t		0.1	
1098	1098	1553	1553	linalool											0.1
1160	1165	1719	1719	borneol							t	0.2	0.2		0.2
1174	1177	1611	1611	terpinen-4-ol	t						0.3	0.2	0.4	0.4	0.8
1201	1189	1706	1706	*α*-terpineol											
1217	1200	1611	1602	*trans*-dihydrocarvone											0.1
1238	1235	1607	1609	thymol methyl oxide	2.6	3.3	2.8	3.1	4.0	4.1	4.2	4.4	5.9	5.7	6.8
1249	1249	1701		thymoquinone						1.0	0.9	0.7	1.7	0.8	2.3
1294	1290	2198	2198	thymol	t						t				0.1
1300	1298	2239	2239	carvacrol	84.0	81.7	82.6	80.0	80.3	78.7	65.7	65.9	61.8	57.9	54.8
1350	1351	1466	1466	*α*-cubebene	0.1	0.1	0.1	t			0.1	0.1	0.1	0.1	t
1378	1376	1497	1497	*α*-copaene	0.1	0.1	0.1	0.1	0.1	0.1	0.1	t	0.1	0.1	0.1
1386	1384	1535	1535	*β*-bourbonene	t										
1416	1418	1612	1612	*β*-caryophyllene	1.7	1.5	1.5	1.9	1.0	1.1	0.7	0.7	0.4	0.4	0.5
1436		1573	1573	*trans*-*α*-bergamotene											

1438	1439	1628	1628	**aromadendrene**	t	0.1	t	0.1	0.1	0.1	0.1	0.1	0.1	0.1	0.1
1454	1454	1668	1670	***α*-humulene**	0.3	0.3	0.3	0.4	0.2	0.2	0.3	0.2	0.2	0.2	0.3
1457	1458	1689	1689	***trans-β*-Farnesene**							t	t			
1478	1477	1704	1704	***γ*-muurolene**	t	t	t	t		t				t	t
1480	1480	1726	1726	**germacrene D**	0.1	0.1	0.1	t	t					t	t
1492		1708	1707	**ledene**	0.2				0.1	0.1	0.1	t		0.1	0.3
1497		1756	1756	**bicyclogermacrene**		0.2	0.2	0.2							
1500		1740	1740	***α*-muurolene**											
1507	1509	1741	1743	***β*-bisabolene**	0.8	0.8	0.6	0.5	0.9	1.0	1.6	1.6	1.3	1.4	1.8
1513	1513	1776	1776	***γ*-cadinene**	t	t	t	t		t	0.1	0.2	0.2	0.1	0.2
1526	1524	1773	1773	***δ*-cadinene**	0.2	0.2	0.2	0.2	0.1	0.2	0.1	0.1	0.1	0.2	0.2
1540		1784	1784	***trans-α*-bisabolene**	t	t	t		t	t					
1558		1984	1984	***γ*-calacorene**					0.1	0.2		t	0.4	0.4	1.0
1577	1576	2152	2150	**spathulenol**	0.1	0.1	0.1	0.2	0.1	0.1	0.2	0.2	0.3	0.2	0.2
1581	1581	2008	2008	**caryophyllene oxide**	0.1	0.1	0.2	0.3	0.3	0.3	0.8	0.9	1.2	1.3	1.8
1608	1606	2202	2071	**humulene-1,2-epoxide**				t	t	t		0.2			0.3
1640		2316	2316	***β*-caryophylladienol**	t			t		t		0.1		0.1	0.2
1686	1683	2229	2229	***α*-bisabolol**											0.1
2115	1949	2603	2622	**phytol**											
				**Monoterpene hydrocarbons**	8.3	10.1	9.9	10.7	10.7	8.7	19.9	18.2	19.7	24.1	15.5
				**Oxygenated monoterpenes**	86.7	85.1	85.4	83.2	84.3	82.8	70.3	70.8	68.7	64.4	63.6
				**Sesquiterpene hydrocarbons**	3.3	3.2	2.9	3.4	2.6	2.9	3.1	3.1	2.9	3.1	4.5
				**Oxygenated sesquiterpenes**	0.2	0.3	0.3	0.4	0.4	0.4	1.0	1.4	1.5	1.6	2.6
				**Others**	1.0	1.1	1.2	1.3	1.4	2.1	3.4	3.2	4.2	3.6	4.8
				**Total identified**	**99.5**	**99.7**	**99.6**	**98.9**	**99.3**	**97.0**	**97.7**	**96.7**	**96.9**	**96.8**	**91.0**

International Journal of
Molecular Sciences

Notes: Drying method (L: lyophilization; D: shade-drying at 4 °C)
Harvest number (1: harvest at the beginning of the month; 2: harvest in the middle of the month)
Retention index (Ri[a]: Retention index calculated on a HP-5MS column; Ri[b]: Retention index on a HP-5MS column from literature [42]; Ri[c]: Retention index calculated on a HP Innowax column; Ri[d]: Retention index on a HP Innowax column from literature [60,61]
t: trace (less than 0.05.%)
Blanks: not detected

References

4. Mouterde, P. *Nouvelle flore du Liban et de la Syrie* ; Distribution Librairie Orientale : Beyrouth, Liban, 1983.
42. Adams, R.P. *Identification of Essential Oil Components by Gas Chromatography/Mass Spectrometry*, 4th ed.; Allured Publ. Corp.: Carol Stream, IL, 2007.
60. De Falco, E.; Mancini, E.; Roscigno, G.; Mignola, E.; Taglialatela-Scafati, O.; Senatore, F. Chemical Composition and Biological Activity of Essential Oils of *Origanum vulgare* L. subsp. *vulgare* L. under Different Growth Conditions. *Molecules* **2013**, *18*, 14948–14960, doi:10.3390/molecules181214948.
61. Khoury, M.; Stien, D.; Eparvier, V.; Ouaini, N.; El Beyrouthy, M. Report on the Medicinal Use of Eleven Lamiaceae Species in Lebanon and Rationalization of Their Antimicrobial Potential by Examination of the Chemical Composition and Antimicrobial Activity of Their Essential Oils. *J. Evid. Based Complement. Altern. Med*. **2016**, *2016*, 2547169.

© 2019 by the authors. Submitted for possible open access publication under the terms and conditions of the Creative Commons Attribution (CC BY) license (http://creativecommons.org/licenses/by/4.0/).
